# Miocene ocean circulation shifted expansive oxygen deficient zones to the Atlantic

**DOI:** 10.1038/s41467-026-73732-7

**Published:** 2026-05-28

**Authors:** Janet E. Burke, Keyi Cheng, Andy Ridgwell, Donald E. Penman, Dalton S. Hardisty

**Affiliations:** 1 Michigan State University, Department of Earth and Environmental Sciences, East Lansing, MI USA; 2 University of California Riverside, Earth and Planetary Sciences Department, Riverside, CA USA; 3 Utah State University, Department of Geosciences, Logan, UT USA

**Keywords:** Marine chemistry, Palaeoceanography

## Abstract

Contemporary observations indicate that dissolved oxygen concentrations are generally declining as global temperatures rise, which has broad implications for carbon cycling and the habitable ranges of marine animals. Here, we use the foraminiferal iodine redox tracer to evaluate the distributions of oxygen deficient zones (ODZs) and adjacent low oxygen water masses in the oceans during the Miocene Climatic Optimum (‘MCO’, 14.7−17 million years ago)—the last time that atmospheric CO_2_ was consistently higher than today. The Pacific lowest oxygen water masses were confined to a narrow latitudinal range of ~10°N-20°S, which is substantially contracted relative to today. In contrast, in the Atlantic, where modern ODZs are minor compared to their Pacific counterparts, our data indicate spatially expansive low oxygen distribution during the MCO. Earth System model simulations provide evidence that the Pacific-Atlantic ODZ seesaw was driven by the very different pattern of ocean circulation and nutrient transport that was induced by an open Central American Seaway. Our findings highlight the key role played by tectonics and ocean circulation, independent of warming, in setting the pattern of ODZs and hence related loci of organic burial and marine habitats.

## Abstract (web summary)

Distributions of expansive low oxygen waters during the Miocene Climatic Optimum greenhouse interval (14.7-17 million years ago) were flipped compared to today, with low oxygen waters engulfing much of the Equatorial Atlantic but contracted in the Pacific.

## Introduction

The Miocene Climatic Optimum (MCO) occurred 14.7–17 million years ago (Ma) and is characterized by a positive carbon isotope excursion (reflecting enhanced carbon burial) as well as temperatures some 3–8 °C higher than today’s^1,2^ (Supplementary Fig. 1). The MCO is the last so-called “greenhouse” climate interval before the establishment of permanent glaciation in the Arctic region during the Pliocene, with a concentration of carbon dioxide (CO_2_) in the atmosphere around 500 ppm^3^—higher than the current value (near 420 ppm) but lower than values of 600 ppm plausible in the coming century under “business as usual” greenhouse gas emission scenarios^4^. As a result, the MCO and other Cenozoic warm periods have attracted considerable interest for their potential as analogs for future CO_2_-driven climate change impacts, including that of warming on ocean oxygen levels^5–8^.

**Fig. 1:**
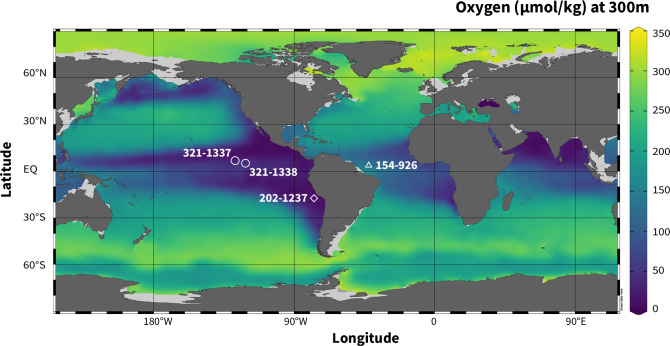
Map of sample localities and oxygen concentration at 300 m depth, with relevant modern sample localities indicated. The site labels represent the IODP or ODP Expedition hyphenated with the Site number designation. This map was generated in Ocean Data View (Schlitzer, Reiner, Ocean Data View, https://odv.awi.de, 2021) from World Ocean Atlas data^12^. IODP Sites U1337 and U1338 are shown as the same symbol, as these sites are merged in Miocene geographical and environmental reconstructions for later discussion and figures.

Rising temperatures affect both the solubility of oxygen (which decreases) and microbial respiration rates that consume oxygen (which increase). These in turn shape the extent and intensity of marine oxygen-deficient zones (ODZs)—sub-surface depth intervals (100–600 m) of the ocean where dissolved oxygen concentrations dip below thresholds for anaerobic respiration. As a result, ODZs are expected to grow as global temperatures rise^9,10^. Today, the largest volume of low oxygen waters is located in the Pacific ODZs, with a much more limited ODZ volume in the Atlantic (Fig. 1)^11,12^. However, recent findings from the Pacific Ocean during the MCO suggest that ODZs may have contracted, not expanded, under warming conditions^5,6^—an inference also made for other Cenozoic warm intervals^7^. Further, while a positive carbon isotope excursion during the MCO has been interpreted to reflect increased organic carbon burial driven by lower ocean oxygenation^13,14^, carbon mass balance approaches instead suggest that MCO warming caused organic carbon burial to be reduced rather than enhanced^15^. Resolving the interpretation of the geological data is critical if we are to draw lessons from the past regarding the impact of higher CO_2_ and ocean warming on dissolved oxygen concentrations and marine ODZs.

The foraminiferal I/Ca proxy provides a snapshot of local redox conditions^16–18^ and offers several distinct advantages in reconstructing past ocean redox conditions, which we leverage in this study. The I/Ca proxy is a measurement of the ratio of iodine-to-calcium in the calcite tests of foraminifera. The oxidized species of iodine, iodate (IO_3_^−^), is the only species of iodine that is incorporated into the calcitic mineral lattice of foraminiferal shells^19^. When oxygen in the water column is near depletion, iodate is reduced to iodide, and the I/Ca ratio recorded in foraminiferal tests falls below 2.3 µmol/mol—a threshold which distinguishes low oxygen water masses from oxygenated water masses^16,20,21^. Importantly, the I/Ca proxy generally tracks the minimum O_2_ in underlying or adjacent waters as opposed to the O_2_ concentrations in waters directly hosting the foraminiferal life cycle. This relationship between I/Ca and dissolved minimum O_2_ in the water column is observed in core-top samples^16^, but the exact timing of iodate incorporation into foraminiferal calcite has yet to be determined^22^ and could be impacted by post-mortem or post-depositional burial processes. This indicates that the foraminiferal I/Ca proxy is an integrated signal across the water column, incorporating subsurface waters above and below low oxygen thresholds^22^. An I/Ca range of <2.3 µmol/mol is herein and commonly assigned to O_2_ values < 75 µM, but this I/Ca-O_2_ threshold relationship is subject to uncertainty (Supplementary Fig. 2)^6,20,23–26^. The proxy nonetheless remains useful because open ocean oxygen concentrations in this range are specific to ODZ-linked water masses, imparting biological stress^27^.

Here, we present new data from foraminiferal I/Ca measurements alongside a series of Earth system model (cGENIE^28,29^) simulations. We measured I/Ca of planktonic foraminifera from four sediment core localities that span the mid-Miocene: IODP Expedition 321, Sites U1337 and U1338 from from the equatorial Pacific; ODP Expedition 202, Site 1237 from the Southeastern Pacific; and ODP Expedition 154, Site 926 from the western tropical Atlantic (Fig. 1; Supplementary Fig. 1). We then analyzed spatial trends in our data and previously published foraminiferal datasets^5,6^ in the context of projected dissolved oxygen distributions.

## Results and discussion

### Geography of Miocene oxygen-deficient zones

**Fig. 2:**
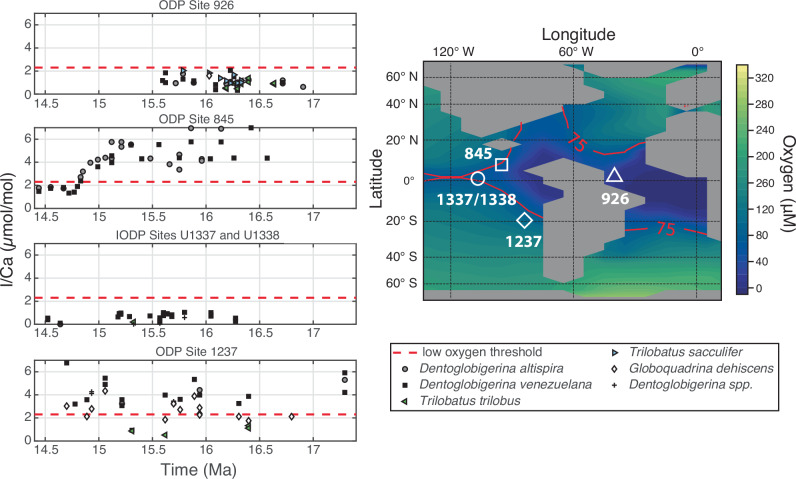
Iodine-to-calcium ratios (I/Ca) of foraminifera from four different mid-Miocene sections. Dashed and solid red lines from the map and figures indicate the I/Ca-ODZ relationship^16^. The inset map shows estimated paleo locations of samples over cGENIE projections of water column minimum oxygen distributions during the mid-Miocene. The site symbols on the map do not correspond to any specific symbols for foraminiferal species in the data figures. Data from ODP Site 845 come from Hess et al.^6^.

Foraminifera from ODP Site 1237—our southernmost Pacific site—were measured between 17.3 and 14.8 Ma. Overall, the I/Ca variability from ODP Site 1237 indicates that the locality was likely near the fringe but not directly within low oxygen waters characterizing an ODZ. Species measured from this site, chosen based on abundance and preservation, were *Dentoglobigerina venezuelana, Dentoglobigerina altispira, Globoquadrina dehiscens*, and, less frequently, *Trilobatus trilobus*. Throughout most of this interval, the average I/Ca values of foraminiferal specimens were above low oxygen thresholds (Fig. 2), but there are species-specific I/Ca differences not observed at other sites. Specifically, *D. venezuelana* and *D. altispira* are elevated, but variable, while *G. dehiscens* oscilates near the low oxygen I/Ca threshold. A smaller pool of samples from *T. trilobus* remains within I/Ca ranges interpreted as representing low oxygen waters. These variable I/Ca MCO observations at ODP Site 1237 are supported in part by similar I/Ca trends observed from an independent ODP Site 1237 study bookending the MCO^30^. The intraspecies variations may represent temporal fluctuations in proximity to low oxygen waters. Interspecies differences may represent variability within depth habit and thus proximity to low oxygen waters or currently uncharacterized species-specific partitioning of iodate. Consistent with this interpretation, the paleolocation of the ODP Site 1237 places it further from the direct site of marginal upwelling during the MCO relative to today^31^. While dissolved iodate is lowest in modern marginal upwelling localities, low iodate (high iodide) waters do persist offshore in the modern Pacific (Supplementary Fig. 3), thus allowing the potential for ODP Site 1237 to track ODZ distribution via foraminiferal I/Ca and increasing its value as a comparison to the modern.

Records from Pacific equatorial IODP Sites U1337 and U1338 were combined to increase temporal coverage of the region, which spans approximately from 14.5 to 16.3 Ma. Foraminifera from these sites consistently measure well below the 2.3 µmol/mol I/Ca threshold, with no discernible inter- or intraspecies temporal trend, which indicates persistent ODZ conditions. Species picked from these samples were also from the intermediate/deep dwelling genera *Dentoglobigerina* and *Globoquadrina*, chosen on the basis of abundance in the samples. Notably, this signal is markedly different from recently published I/Ca data from the nearby, but slightly northward, ODP Expedition 138, Site 845 (Fig. 2)—also measuring *D. venezuelana* and *D. altispira*—that reflects a well-oxygenated water column until shifting to ODZ conditions around 15 Ma^6^.

Together, these results indicate that while there was a persistent equatorial Pacific ODZ present throughout the MCO, the spatial range of the Eastern Pacific ODZ was significantly restricted compared to its modern extent. The most intense ODZ conditions were concentrated near the equator in the Eastern tropical Pacific and restricted to a maximum extent of ~10°N-20°S. In the modern Eastern Pacific, a contiguous midwater low oxygen zone with dissolved oxygen levels below 50 µmol/kg stretches from ~30°N to ~40°S and far west along the tropical latitudes (Fig. 1). These modern low O_2_ waters correspond to the lowest water column iodate concentrations^32–34^.

Foraminifera from the western Atlantic (ODP Site 926) also record I/Ca values that indicate that the site had an expanded ODZ throughout the observed interval, ~15.5–16.9 Ma. A diversity of species was measured from these samples, all with low I/Ca values, including the surface-dwelling *Trilobatus sacculifer* and *T. trilobus*, as well as some of the intermediate/deep dwelling *D. venezuelana* and intermediate dwelling *G. dehiscens* and *D. altispira*. Importantly, *D. venezuelana* and *D. altispira* were also measured at each of the other sites and show the potential to have elevated I/Ca at ODP Sites 1237 and 845, indicating that the low values at ODP Site 926 are not related to a species-specific vital effect. In addition, foraminiferal I/Ca in the modern Atlantic shows a similar relationship to O_2_ as other ocean basins (Supplementary Fig. 2), supporting that our observations reflect foraminiferal proximity to low oxygen waters as opposed to a currently uncharacterized Atlantic-specific basinal effect on foraminiferal I/Ca.

Evidence for very low oxygen levels in shallow and midwater strata in the western Atlantic is surprising, given the geography of ODZs in the modern Atlantic. Today, more restricted ODZ conditions are found in the Atlantic compared to the Pacific and are mostly limited to the southwestern coast of Africa at the upwelling Benguela current (Fig. 1). The modern western Atlantic and Caribbean are generally well oxygenated throughout the water column and Atlantic ODZ dissolved iodate concentrations generally do not reach the low levels observed in the more reducing waters of the Pacific ODZs (Supplementary Fig. 2)^35,36^.

The presence and spatial extent of Atlantic ODZ expansion inferred here from I/Ca is further constrained by independent observations. For example, the presence of *Globorotaloides hexagonus* has been demonstrated to be most abundant in areas that have strong midwater ODZs and thus its presence in ancient sediments has been applied as an indicator of the presence of low oxygen water column conditions during burial^30,37,38^. At ODP Site 926, we observed the presence of specimens of *G. hexagonus* in every one of a subset of nine samples examined, although those specimens were not included in the I/Ca measurements (Supplementary Fig. 4; Supplementary Table 1). Further paleontological evidence for Miocene ODZ conditions spanning the Atlantic—though not all of these observations overlap with the MCO—includes periods with ostracod and benthic foraminiferal fauna characteristic of anoxic habitats^39–42^. Other studies provide some constraints on the sources and spatial extent of ODZ water masses in the Atlantic. Specifically, a site adjacent to ODP Site 926 has evidence for low productivity^43^, which may indicate that low I/Ca in this region is the result of preservation and transfer of low iodate produced elsewhere in the ODZ^44^. Further, studies of N isotopes tracking denitrification in the southern Atlantic ODP Expedition 72, Site 516, do not show evidence for low oxygen conditions^5^. Since iodate reduction and denitrification commonly overlap in modern^33,45^ (Supplementary Fig. 3) and paleorecords^6,30^ this difference between the equatorial and a more southerly site could provide some constraints on the southern extent of the low O_2_ water masses in the Atlantic.

Importantly, the comparison of the nearby IODP Sites U1337 and U1338 provides a diagenetic control, as previous work has noted exceptional preservation in IODP Site U1338 compared to IODP Site U1337 during our study interval^46^, which was also confirmed here. Observed preservational differences have been linked to the relatively higher depositional rates at IODP Site U1338, limiting the residence time of foraminifera within sediment layers with more corrosive diagenetic conditions^46^. Despite the differences, both cores maintain low I/Ca during the MCO, which is also observed in post-MCO records from these same sites^30^. Although some samples from the Pacific IODP Sites U1337 and U1338 and many from ODP Site 1237 had low planktonic foraminiferal abundance, we do not see indications that poor calcite preservation exerts a major influence on overall trends in I/Ca. Samples from IODP Site U1338, which returned some of the lowest I/Ca values, have the best preservation of the samples analyzed^46^. Samples from ODP Site 1237, which had more frequent markers of poor preservation (see “Assessing Diagenetic Recrystallization” in “Methods”; Supplementary Fig. 5), returned some of the highest I/Ca values. Our SEM examinations of target specimens did not return evidence for extensive recrystallization or dissolution from the sites with the lowest I/Ca. Further, where consistently low I/Ca values were seen, the trend was consistent among different species and samples throughout the time series (Fig. 2). Thus, we do not find evidence for a systematic relationship between calcite preservation and I/Ca values.

**Fig. 3:**
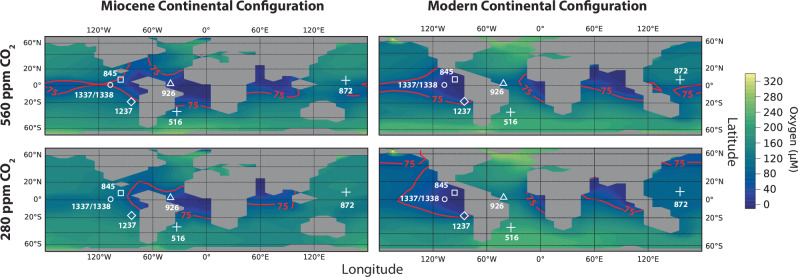
Model sensitivity analyses of minimum oxygen concentrations in relationship to modern versus Miocene continental configuration and atmospheric [CO_2_]. The red line marks the oxygen concentration threshold leading to lower I/Ca ratios indicative of low oxygen conditions^16^. The markers show relevant sites from the main text at their respective MCO locations (including for the right 2 panels with modern continental configuration). ODP Sites 516 and 872 (crosses) were not measured for I/Ca, but have paleoreodox proxy constraints consistent with oxic conditions^5,6^.

To help interpret the I/Ca data in terms of Miocene oxygen patterns and mechanisms, we modeled oxygen and iodate distribution under Miocene climate and paleogeographic boundary conditions (Figs. 2 and 3)^47^ using the cGENIE framework—an Earth system model of intermediate complexity integrating physical and biogeochemical oceanographic processes^28,29^. Of particular relevance here, our Miocene boundary conditions included elevated atmospheric CO_2_ (560 ppm) compared to modern^3^, and an open Central American Seaway (CAS) connecting the Pacific and Atlantic Oceans at low latitudes.

Model simulations in cGENIE support an expanded Atlantic ODZ, with a low oxygen zone that engulfs the entire equatorial and tropical South Atlantic, which would be necessary in order to explain our I/Ca results from ODP Site 926 in the western Atlantic. Additional Atlantic and Pacific sites with previously published paleoredox constraints indicating well-oxygenated conditions (ODP Expedition 144, Site 872, and ODP Site 516)^5,6^ are notably in locations that are on the margins of or well outside of the severe ODZ boundaries modeled in this study (Figs. 2 and 3).

Our sites constrain the ODZ latitudinal boundaries in the Pacific. Our modeling results show a Pacific ODZ that is constricted during the MCO, but that extends into the open ocean at low latitudes, which is consistent with low I/Ca values from IODP Sites U1337 and U1338 in our data. IODP Sites U1337 and U1338 and ODP Site 845 are geographically close together but show highly contrasting I/Ca ratios during the early MCO (but are both more consistent as being within the ODZ in the latest MCO). Given an unlikely role from diagenesis, we interpret this contrast to indicate that the ODZ margin is located between these 2 sites. Importantly, the model results for dissolved O_2_ generally confirm that the ODZ boundary extends through this region (Fig. 2).

The modeled iodate concentrations indicate that the iodate-reducing region within the Pacific ODZ is more constricted relative to the modeled low O_2_ region and does not extend westward to IODP Sites U1337 and U1338, where low I/Ca is observed (Supplementary Figs. 6 and 7). That our modeling does not reproduce the low iodate region relative to that identified via I/Ca might simply be a consequence of the model’s relatively low spatial resolution (10° in longitude can ca. 5° in latitude close to the Equator) (e.g., see Cheng et al.^28^ for a discussion). Another possible explanation for the difficulty in resolving the sharp spatial I/Ca gradient between the IODP Sites U1337 and U1338 and ODP Site 845 via the model iodate is uncertainty in our understanding of the iodate-O_2_ relationship in modern settings. This is particularly relevant along ODZ boundaries^21^. For example, modern observations indicate that higher iodate concentrations are present before similar increases in [O_2_] along the ODZ margins, including both laterally and at the lower oxycline of the ODZ. Instead of a strict linear relationship, iodate-oxygen can be best explained by active iodate reduction constrained to productive, marginal upwelling zones hosting denitrification, followed by subsequent internal-ODZ transport and preservation of these low iodate waters beyond regions of active iodate reduction (Supplementary Figs. 2 and 3). Iodate concentrations eventually increase at these offshore ODZ margins prior to similar O_2_ increases because of the combination of iodide oxidation and mixing with adjacent high iodate water masses (Supplementary Fig. 3)^32,33,45,48^. Thus, while our data-model comparison generally supports the observations of a restricted Pacific ODZ relative to today, there is uncertainty in resolving the modeled ODZ boundary at the high spatial resolution of our foraminiferal I/Ca data.

### An oceanographic gateway control on ODZs

The question of how marine ODZs respond to conditions of elevated CO_2_ and global temperatures is important for resolving predictions for the future of the oceans as warming continues. Continental configuration and its impacts on ocean circulation patterns are also predicted to be a major driver of ocean oxygen levels over the Phanerozoic^49^. In addition to elevated CO_2_, the CAS was completely or at least mostly open during the Miocene, allowing for open exchange between the Pacific and Atlantic basins. To test the likely relative contributions, if any, of CO_2_ and warming versus continental configuration on ODZ geography, we performed model sensitivity analyses under conditions of CO_2_ and relative opening of the CAS that spanned a gradient from the modern to Miocene^47^.

These sensitivity analyses combined different permutations of climate and continental configurations (Fig. 3 and Supplementary Fig. 9), showing that when the CAS is open, the Pacific ODZ is reduced and restricted to low latitudes, while a large ODZ consumes the tropical Atlantic. When atmospheric pCO_2_ is 280 ppm (pre-industrial), the Pacific ODZ nearly disappears, with oceanic oxygen concentrations consistently above the threshold value that could drive iodate reduction. Notably, in this reduced CO_2_ scenario, the Atlantic is still characterized by an intense, basin-spanning tropical ODZ that encompasses the vicinity of ODP Site 926. Elevating CO_2_ further strengthens the Atlantic ODZ, which then nearly covers the entirety of the tropical latitudes.

When combined with our I/Ca data, the model results support Miocene-specific ocean circulation as the primary driver of this Atlantic-Pacific ODZ seesaw. The Atlantic ODZ is intensified under higher CO_2_ expected of the Miocene but remains even at pre-industrial modern CO_2_ levels (Fig. 3). Further, the ODZs do not appear particularly sensitive to changes in the strength of deep water ventilation via the Atlantic Meridional Overturning Circulation (AMOC) unless completely collapsed, in which case the tropical Atlantic ODZ spreads further northward (Supplementary Fig. 8). Today, the Atlantic intermediate waters are more recently ventilated, and thus higher in O_2_ and lower in nutrients, than that of the Pacific^50^. Rather, it is the openCAS in the Miocene that drives the flipped Pacific-Atlantic ODZ relationship, reducing the intensity of Pacific upwelling and providing a younger O_2_ supply in Eastern Pacific subsurface waters sourced from the Western Pacific^51^.

We also evaluated the impacts of other Miocene-specific differences in paleogeography compared to today on ODZ distribution (Supplementary Fig. 9). For example, the opening of the Drake Passage continued since the MCO, which impacts the Antarctic Circumpolar Current^52^. In simulations of evolving paleogeography since the mid-Miocene under forced open and closed CAS, the opening of the CAS has the clearest impact on Atlantic and Pacific ODZ distribution (Supplementary Fig. 9). Previous models also support Pacific oxygenation and an increase in Atlantic oxygen consumption if the CAS is open even in an otherwise modern ocean, but our Miocene-specific simulations indicate the potential for a much more extreme Atlantic ODZ than previously recognized. For example, in our simulations, intermediate water ventilation in the North and South Atlantic does not reach the mid-Atlantic and ultimately restricts the outflow of the mid-Atlantic’s CAS-sourced subsurface water supply, further concentrating nutrients and exacerbating the Atlantic ODZ (Supplementary Figs. 11–14). This observation is consistent with the lack of denitrification signal (expected in low oxygen waters) found at the more southerly Atlantic ODP Site 516^5^, which is outside of the low oxygen water masses modeled in our study (Fig. 3).

**Fig. 4:**
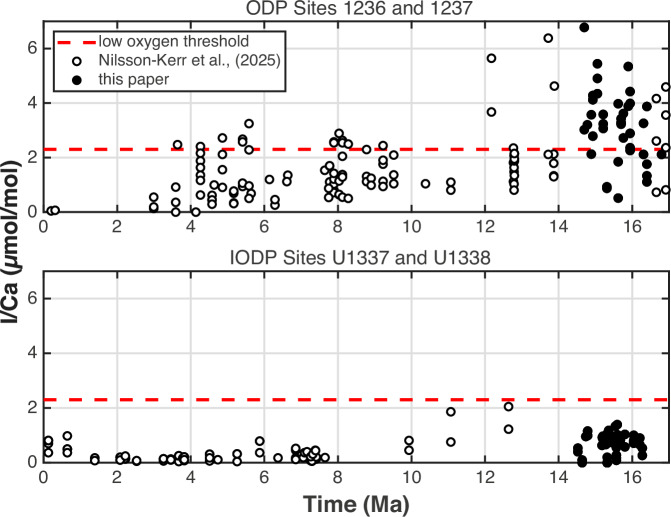
Compilation of Eastern Pacific I/Ca data. Top Panel: Measurements from Nilsson-Kerr et al.^30^, from IODP Site U1338 plotted with data from this study from IODP Sites U1337 and U1338; Bottom Panel: Measurements from Nilsson-Kerr et al.^30^, from ODP Sites 1236 and 1237 plotted with data from this study from ODP Site 1237.

The evolution of the closing of the CAS might be constrained in part by longer temporal records of ODZ distribution^51^ (Fig. 4). Combined with previous records of younger intervals, our I/Ca data demonstrate that Site IODP U1337 has been located within an ODZ region since the MCO. ODP Site 1237 evolved to ODZ conditions starting after the MCO in the late Miocene^30^, which is consistent with the timing of reconstructed shifts from lower to elevated nutrients at this site^31^. ODP Site 1237 was further southwest from the Pacific upwelling zone and ODZ during the MCO than it is today^31^, and thus this evolution to ODZ conditions likely represents some combination of ODZ expansion as well as plate tectonics. Although there is some evidence that the CAS closure was underway around the time of the MCO^53,54^, the timing of the expansion of Pacific ODZ conditions supports that the CAS did not close to isolate Pacific and Atlantic ODZ water masses until at earliest the Late Miocene^30,51^.

### Implications for ancient climate change analogs

Our data and model suggest a spatial distribution of ODZs flipped between the MCO and today, as opposed to a globally consistent weakening trend in response to higher atmospheric CO_2_ and warming. Although Pacific ODZs were contracted during the MCO, the Atlantic may have been the locus of an expansive mid-depth ODZ that grew in intensity at the height of Miocene warming. This switch in Atlantic-Pacific ODZ patterns during the MCO relative to today was largely a consequence of the continental configuration and hence the large-scale pattern of ocean circulation at the time. Our models and data emphasize an important role of intermediate water ventilation in driving ODZ redistribution relative to today. Ocean circulation variations may thus partially explain why some individual regions have been documented as having intensified or weakened ODZs^6,13^ while independent studies of carbon burial^15^, productivity^43^, and bottom water redox^15,55,56^ indicate overall global conditions similar to the modern.

Our joint Atlantic and Pacific observations and models indicate that interpretations of warming-induced ODZ contraction drawn from the Pacific during the MCO^6,13^ may be incomplete analogs for comparison to ODZs during modern and future warming. Ultimately, while Cenozoic warm periods—including the MCO—can inform future impacts of continuing carbon emissions and warming, it is essential to account for differences in continental configuration and ocean circulation that impact local and global ODZ distribution.

## Methods

### Foraminiferal sample preparation and measurement

Sediment samples were washed over a 63 µm sieve with deionized water, drained through filter paper, and dried in a 45 °C drying oven. This process was repeated as necessary to fully disaggregate the sediment and remove the fine fraction. Twenty to thirty specimens were picked from the 300–425 µm and 425-650 µm sieved size fractions for each sample analysis^57^.

Target species for analysis were chosen to capture surface-dwelling and thermocline-dwelling species from each sample where available. Species from the genus *Dentoglobigerina* were abundant in all samples, particularly *Dentoglobigerina altispira* and *Dentoglobigerina venezuelana*. These species are understood to be deep intermediate to deep dwelling (thermocline or sub-thermocline) based on their δ^13^C and δ^18^O profiles. Additionally, we measured *Globquadrina dehiscens*, an intermediate depth dwelling species that was abundant in some samples (Supplementary Table 1).

Picked specimens were lightly crushed between glass slides and rinsed in ultrapure water and sonicated for 7-10 repetitions to remove clay particles, then washed with a buffered hydrogen peroxide solution to remove organic matter (Method from Barker et al.^48^ as modified by Zhou et al.^17,58^). Cleaned samples were dissolved in a 3% solution of nitric acid and sonicated on the same day of analyses and diluted in a 0.5% tertiary amine solution (Inorganic Ventures UNS-2B) within 30 min of sample dissolution. Elemental ratios of samples were measured on a Thermo-Fisher iCAP Triple-Quadrupole ICP-MS at Michigan State University. Calibration standards were made fresh on the same day of each analytical run. Each standard contained 50 ppm Ca, and samples were diluted to 50 ppm Ca to matrix match the standards. A fresh JCp-1 (Japanese Coral – Porites) reference standard—dissolved and diluted according to the same approach as for unknowns—was monitored at the beginning of each analytical run to assure that the I/Ca value fell within the known range for the standard^59^. The JCp-1 values across individual ICP-MS runs from this study (*n* = 13 over nearly 3 years, Supplementary Table 2) were 3.96 ± 0.19 µmol/mol (1standard deviation), which directly overlaps with the values of previous studies^59^. JCp-1 and a calibration standard were also included throughout each analytical run, following every 5–7 unknowns, and used to monitor drift in I/Ca values.

For most samples, individual pools of 20–30 specimens of the same species were measured for multiple species. The species-specific data are shown in Fig. 2. In addition, for every sample with sufficient availability of target species (42 of the 49 total depths analyzed), additional measurements were made of separate pools of 20–30 specimens. Replicates were also measured across multiple analytical runs. The sometimes higher range observed at higher I/Ca ratios (e.g., ODP Site 1237) is consistent with previous studies^57^ and integrates combinations of analytical uncertainty, species-specific differences, as well as variations between individual foraminifera from each replicate pool (Supplementary Table 1).

### Assessing diagenetic recrystallization

The conditions that are present in upwelling zones and intense ODZs can hinder the pristine preservation of carbonate foraminiferal tests. Poorly preserved foraminifera, specifically those that have undergone post-depositional dissolution and/or recrystallization, can lose their original trace metal and isotopic signatures, instead reflecting porewater conditions^60^. This could be especially important for I/Ca measurements, as porewaters are typically depleted in oxygen and iodate^61^. Diagenetic overprinting could create a false ODZ signal if the proxy is indeed sensitive to diagenesis. Indirect evidence presented in Zhou et al.^18^ comparing the I/Ca values of deep and shallow-dwelling planktonic foraminifera suggests that I/Ca is not sensitive to diagenetic alteration. We sought to test this further by sampling from two adjacent localities in the Eastern Tropical Pacific (IODP Sites U1337 and U1338) that have been reported to have different preservation states^46^. Selected samples from these localities that are close in estimated age were picked for overlapping species and imaged using a scanning electron microscope at the MSU Center for Advanced Microscopy to compare surface texture and look for evidence of recrystallization and dissolution^60,62^. We found that the samples examined from IODP Site U1337 varied widely from poor to good preservation, while the samples from IODP Site U1338 were more uniformly good or at least moderate (Supplementary Fig. 5; Supplementary Table 3). Despite these differences, the I/Ca values were uniformly low at both sites. Further, ODP Site 1237, which had the worst preservation in the samples we analyzed, generally produces higher I/Ca values than any of the other sites. The samples from ODP Site 926 displayed moderate preservation markers and produced low I/Ca values consistently. In summary, we do not find evidence for a systematic relationship between calcite preservation and I/Ca values.

### Sample age and paleolocation determination

Ages for samples from IODP Sites U1337 and U1338 and ODP Site 1237 were determined using previously published resources for the localities. For samples from IODP Sites U1337 and U1338, we followed the age model used in Holbourn et al.^1^ for adjacent samples from IODP Site U1337. Age estimates for ODP Site 1237 are from Holbourn et al.^63^ estimates for the same locality. Recent updates to these established age models from IODP Sites U1337 and U1338 and ODP Site 1237 from Holbourn et al.^64^ were also incorporated. For ODP Site 926, the age of samples was determined from the age model used in Sosdian et al.^65^ (SsupplementaryTable 4). Miocene sample localities were estimated using the Paleolocation Mapping Service website (http://www.paleolocation.org/^66^). Coordinates provided in Supplementary Table 6.

### cGENIE model details

We adopted bathymetry, climate, primary productivity, continental configuration, and remineralization following Crichton et al.^52^ and Boscolo-Galazzo et al.^34^, and iodine cycling parameters calibrated by Cheng et al.^28^. All the model simulations in this manuscript were based on the “muffin” release of the cGENIE Earth System Model of intermediate complexity (EMIC) (v0.9.54) with an assigned 10.5281/zenodo.13376310. Our model simulations used the equal area 36 ×  36 grid, which equates to 10° in longitude and latitude increments from 3° near the equator to 20° near the pole, plus logarithmically spaced 16 vertical levels from sea level to 5000 m depth below the sea surface^67^. The ocean model is coupled to a 2D energy-moisture-balance model and a 2D dynamic-thermodynamic sea-ice model^68,69^. Comparing to high-resolution Earth System Models, such intermediate complexity models require less CPU hours for long-term simulation (10,000 model years/24 h), therefore suitable for paleoceanographic studies as well as sensitivity analyses of large ensembles. Each model simulation was run for 10,000 years from the initial state (0% sea ice, 5 °C SST, 33.9PSU). To determine the factor (climate, ocean circulation) that most significantly controls the ocean redox pattern, sensitivity analyses were run by varying bathymetry through the last 15 Ma as well as open/closure of the CAS, CO_2_ levels, and salinity (Supplementary Fig. 9) to allow the system to reach a steady state. Only one time slice was saved at the end of each run.

To simulate the impacts of the strength of the AMOC on ODZ distribution, we applied the concept of “salinity flux adjustment” (FwF) as proposed and calibrated by Crichton et al.^52^ and Boscolo-Galazzo et al.^47^. The FwF impacts the North Atlantic surface salinity, which impacts thermohaline circulation related to the AMOC. As described in Crichton et al.^52^, higher FwF values produce higher salinity surface water in the North Atlantic, which increases downwelling and transport from the surface to the deep. Notably, in Crichton et al.^52^, the salinity flux adjustments determined to best fit paleo proxy data were calibrated under a model study that used pCO_2_ for the Miocene at higher ranges (up to 1120 ppm) than that evaluated in our study, and Boscolo-Galazzo et al. found that at 560 ppm, the best fit FwF for the mid-Miocene was 0 Sv. For our model, we chose a maximum pCO_2_ value of 560 ppm based on widely accepted pCO_2_ estimates for the MCO^3^. In addition, the earlier calibration of Crichton et al.’s work did not include the temperature-dependent remineralization scheme added by Boscolo-Galazzo et al.^47^ and applied here and in the recent iodine cycle model calibration in cGENIE in Cheng et al.^28^. For these reasons, we performed a sensitivity analysis to determine the impact of the salinity flux adjustment under the pCO_2_ from our study (Supplementary Figs. 8 and 10), including FwF ranging from 0 to 0.4. For context, the modern FwF used in cGENIE is 0.32^52^. Our sensitivity analysis indicates that ODZ distribution is not sensitive to non-zero salinity flux adjustment values (Supplementary Figs. 8 and 10). When the FwF is set to 0 Sv, the AMOC is severely impacted, and the tropical Atlantic ODZ spreads northward. In the simulations for the main text Figs. 2 and 3, we used a salinity flux adjustment of 0.1 Sv.

We tested the relative impacts on ODZ distribution of the closing CAS versus the tectonic transformation of other ocean basins since the mid-Miocene (Supplementary Fig. 9). These include the widening of the Drake Passage as South America moved northward, the restriction of the Indonesian Seaway as Australia moved northward, and the disappearance of the Tethys Sea with the northward movement of Africa^52^. Following Crichton et al., we tested these impacts on ODZ distribution through model simulations under evolving paleogeographies from 15 Ma to present under conditions both forcing the CAS open and CAS closed. The model runs demonstrate that the CAS and subsequent closing has the largest impact on Atlantic and Pacific ODZ distribution.

The iodine cycle in cGENIE is described in detail in Cheng et al.^28^, but is summarized below. Iodine cycling in the model consists of 3 reservoirs, including dissolved iodate, iodide, and both iodate and iodide incorporated into particulate and dissolved organic matter. While iodine is distributed via physical circulation according to properties in the model as described above, there are 4 processes that impact biogeochemical cycling of iodine: (1) Iodate reduction in the water column. Based on model calibrations in Cheng et al.^28^, this reduction process best simulated modern iodine distribution when set as a threshold relative to oxygen. In the model, when O_2_ is below 10 µmol/kg, iodate is quantitatively reduced to iodide. (2) Iodide oxidation to iodate, which is described in more detail in the following paragraph. (3) photosynthetic iodate incorporation and internal reduction to iodide. (4) The release of biologically assimilated iodide during the remineralization of particulate and dissolved organic matter.

We modeled the distribution of iodate to compare with the modeled oxygen and our I/Ca data (Supplementary Figs. 6 and 7). The iodine cycle was modeled under three parameterizations (Supplementary Fig. 6) found to provide the best replication of the modern iodine distribution (World Ocean Atlas-tuned parameterizations in Table 2 of Cheng et al.^28^). The three parameterizations represent alternative iodide oxidation mechanisms, while iodate reduction in all three cases occurs quantitatively once local [O_2_] is <10 µmol/kg (i.e., “threshold”). Iodide oxidation parameterizations include: (1) iodide oxidation according to “lifetime,” where oxidation to iodate occurs ubiquitously according to first-order kinetics, regardless of ambient [O_2_] (Supplementary Fig. 6A). This is analogous to that used in Lu et al.^22^, but updated based on modern tuning from Cheng et al.^28^. (2) Iodide oxidation according to “Fennel,” where oxidation to iodate occurs as a function of ambient [O_2_] following Michaelis-Menten kinetics, analogous to ammonia reoxidation in cGENIE^70^ (Supplementary Fig. 6B). This oxidation scheme considers overlaps between iodine and nitrogen cycling zones with ODZs (e.g., Supplementary Fig. 3). (3) Iodide oxidation according to “reminO2lifetime,” where oxidation to iodate occurs during O_2_ consumption during organic remineralization, similar to ammonia oxidation during organic remineralization in cGENIE^71^ (Supplementary Fig. 6C). This oxidation scheme considers recent observations that iodide oxidation to iodate may be catalyzed by ammonia-oxidizing bacteria^72^.

Notably, all 3 parameterizations were found to have similar statistical fits in replicating the modern iodine cycle, but “Fennel” and “reminO2lifetime” performed slightly better in a calibration comparing modeled iodate and observed I/Ca ratios from inorganic calcite from pre-OAE-2^28^. Note that the darkest blue/purple zones (main text Figs. 2 and 3) overlap with the lowest [O_2_] concentrations <10 µmol/kg and are locations where active iodate reduction is occurring in the model. Beyond these zones, low iodate concentrations represent transport and preservation of low iodate water masses formed in the ODZs, as well as some impacts from iodate reduction during phytoplankton growth, also represented in the model^28^.

## Supplementary information

Supplementary Information

Transparent Peer Review file

## Data Availability

The I/Ca data generated in this study have been deposited in the PANGAEA database under accession code 10.1594/PANGAEA.974710. The I/Ca data generated in this study are provided in the Supplementary Information.

## Code availability

The code for the version of the “muffin” release of the cGENIE Earth system model used in this paper is tagged as v0.9.57, and is assigned a 10.5281/zenodo.14366137. Configuration files for the specific experiments presented in the paper can be found in the directory: genie-userconfigs/PUBS/submitted//Burke_etal.2024. Details of the experiments, plus the command line needed to run each one, are given in the readme.txt file in that directory. All other configuration files and boundary conditions are provided as part of the code release. A manual detailing code installation, basic model configuration, tutorials covering various aspects of model configuration, experimental design, and output, plus the processing of results, is assigned a 10.5281/zenodo.1407657.
