# Supplementary material for: Miocene ocean circulation shifted expansive oxygen deficient zones to the Atlantic
Source: Nat Commun. 2026 May 28;17:5935. doi: 10.1038/s41467-026-73732-7 (PMC13341793; doi:10.1038/s41467-026-73732-7)
Supplement: Supplementary file 1 — Supplementary Information [file 41467_2026_73732_MOESM1_ESM.pdf]

Supplemental Figures and Tables

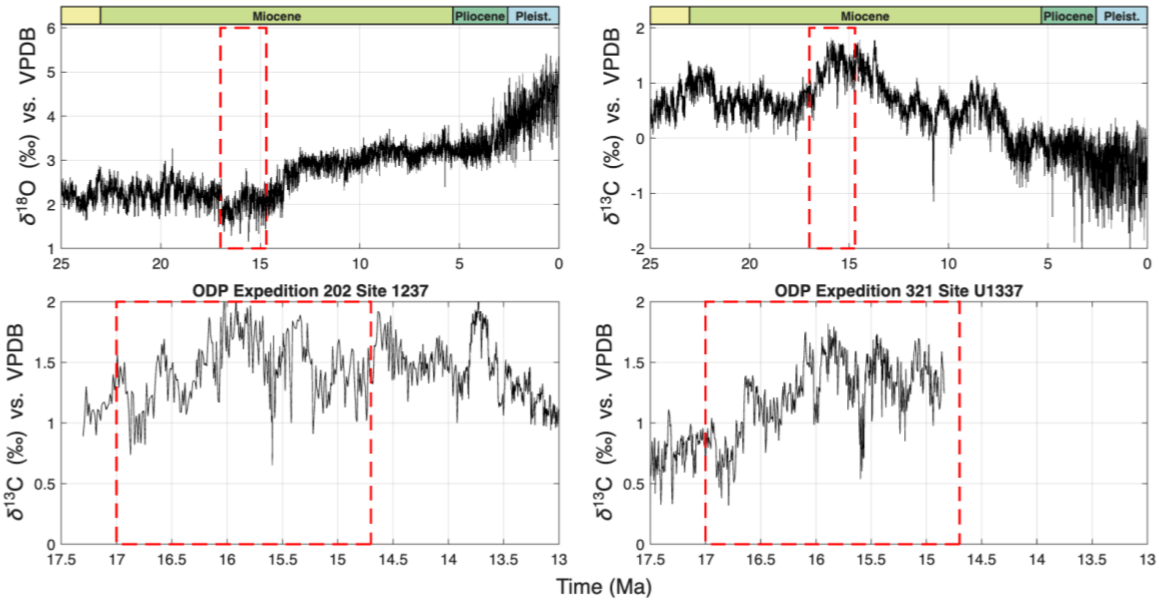

Supplementary Figure 1. Stable Isotope Records of the Miocene Climatic Optimum: Cenozoic $\boldsymbol{\delta}^{13}$C and $\boldsymbol{\delta}^{18}$O isotope records from Westerhold et al.[1] (Top row). Site specific $\boldsymbol{\delta}^{13}$C records for IODP Site U1337 are from Holbourn et al.[2] and ODP Site 1237 are from Holbourn et al.[3] (bottom row). The red box outlines the Miocene Climatic Optimum and Monterey Excursion intervals.

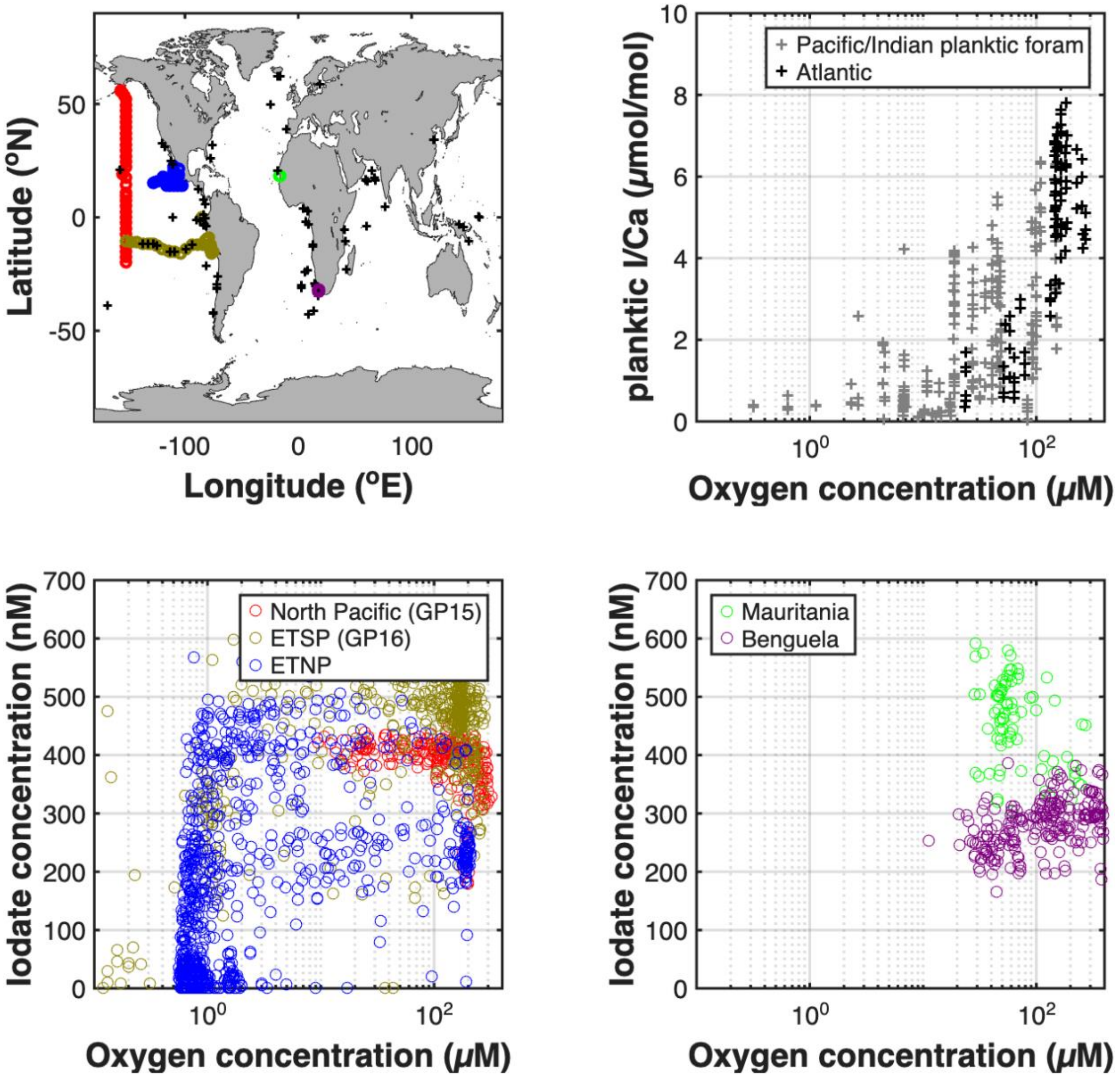

Supplementary Figure 2. Modern Pacific and Atlantic iodate and I/Ca distributions. Top left: Locations of data for water column and planktic foraminifera data in subsequent panels. Top right: Compilation of core top planktic I/Ca ratios relative to minimum water column oxygen concentrations. Figure includes data from et al.[4], Zhou et al.[5]; Lu et al.[6]; Lu et al.[7], and Hess et al.,[8]. Bottom Row: Comparison of water column iodate concentrations and dissolved oxygen concentrations for Pacific versus the Atlantic (left and right, respectively). North Pacific data from Moriyasu et al.[9]. ETSP from Rapp et al.[10] and Cutter et al.[11]. ETNP from Moriyasu et al.,[12] and Rue et al.[13]. Mauritania from Rapp et al.[14]. Benguela from Chapman et al.[15].

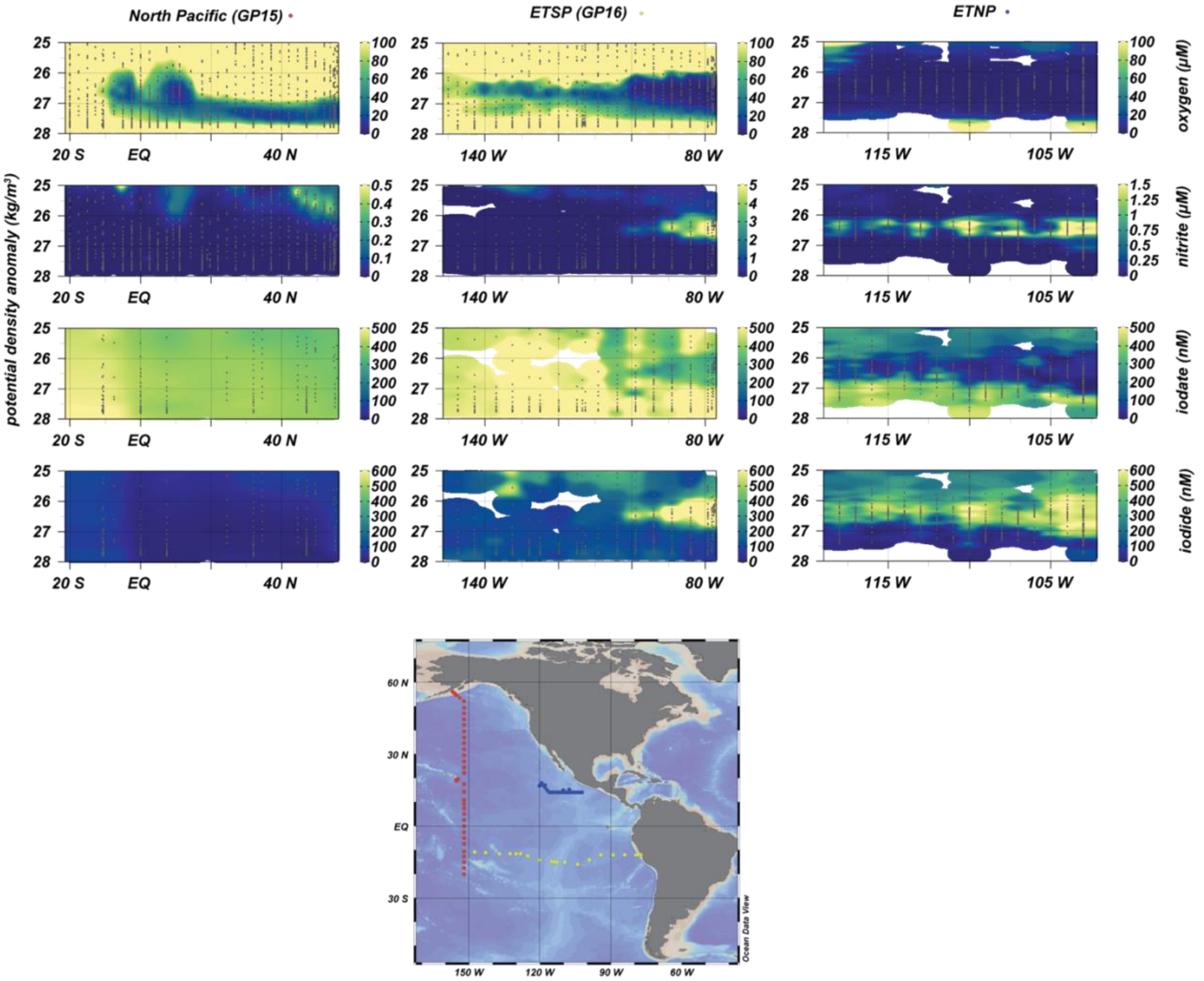

Supplementary Figure 3. Iodate distributions in Pacific oxygen deficient zones. Distributions of oxygen (top row), nitrite (second row), iodate (third row) , and iodide (fourth row) measured along transects in the Pacific Ocean (last row). North Pacific data from Moriyasu et al.[9] and GEOTRACES IDP 2017[16]. ETSP from Cutter et al.[11]. ETNP from Moriyasu et al.[12]. Note that the y-axis shows the potential density anomaly, not depth. In the meridional transects of the ETSP and ETNP, note that the lowest iodate waters overlap with high nitrite waters, and that iodate increases both with depth and laterally even as oxygen concentrations remain low. For the North Pacific latitudinal transect, the ODZ is intersected far offshore from its source along the North American coast, and no low iodate region is observed. Figure created in Ocean Data View (Schlitzer, Reiner, Ocean Data View, https://odv.awi.de, 2021).

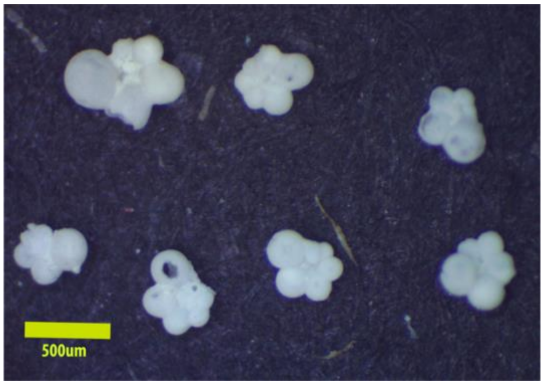

Supplementary Figure 4. *G. hexagonus* specimens: Example of specimens of *Globorotaloides hexagonus* from ODP Expedition 154 Site 926B Sample 33X-2W-103.

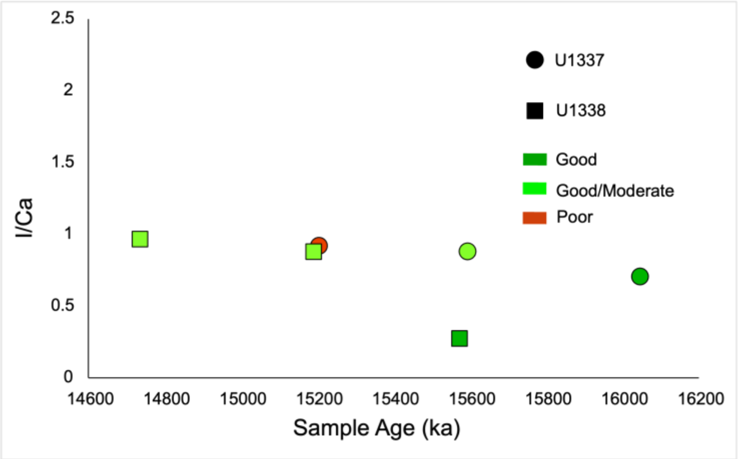

Supplementary Figure 5. Foraminiferal Preservation and I/Ca: Comparison of I/Ca values measured in foraminifera from diagenetically variable adjacent localities IODP U1337 and U1338. Preservation in these samples ranges from Good (dark green) to Good/Moderate (light green) to Poor (red).

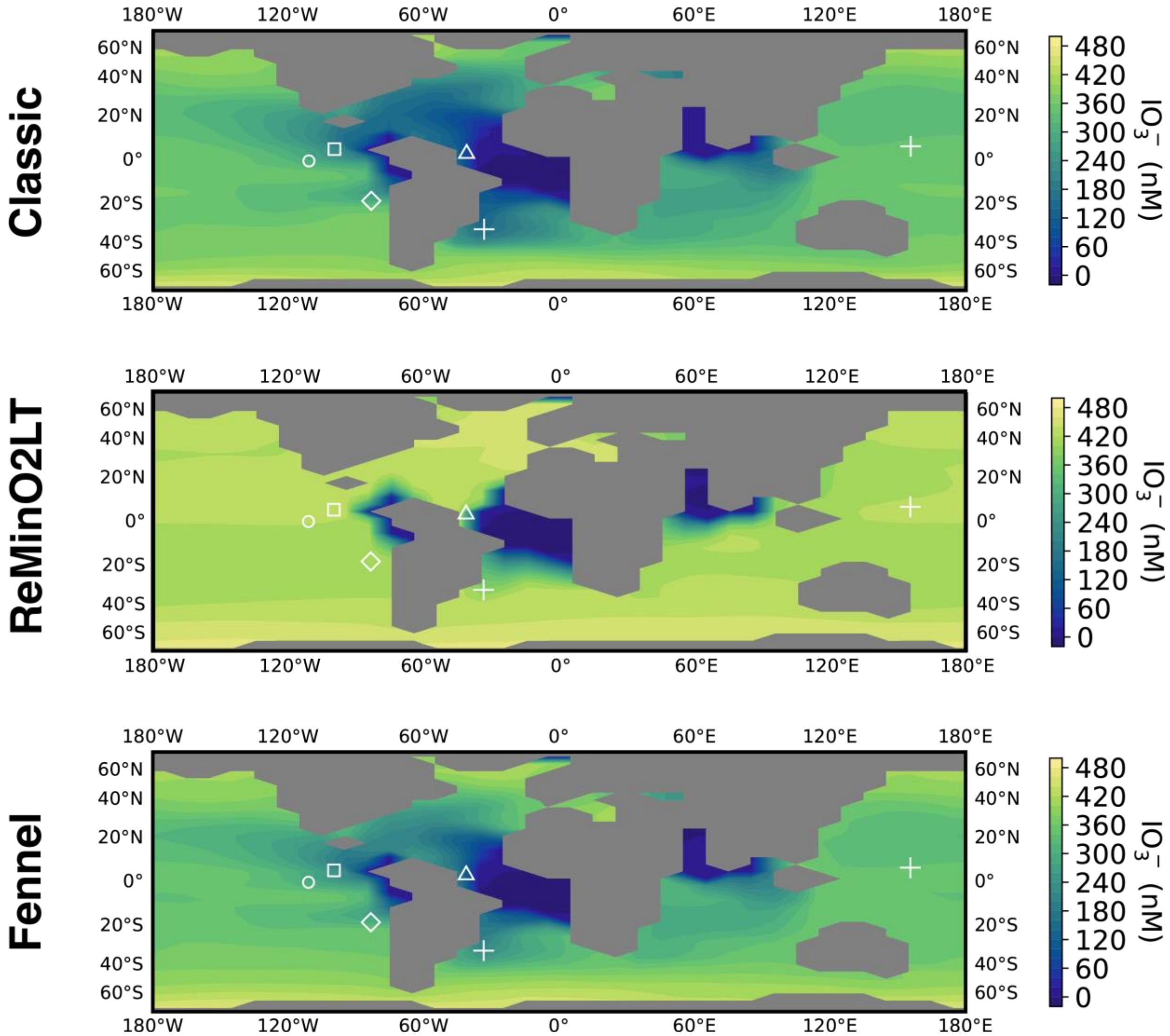

Supplementary Figure 6. Modeled Miocene iodate distributions. Distributions of iodate modeled under the same 560 ppm CO_2 and Miocene continental configuration shown for oxygen in main text Figures 2 and 3. The iodine cycle was modeled under 3 parameterizations found to provide the best replication of the modern iodine distribution (World Ocean Atlas-tuned parameterizations in Table 2 of Cheng et al.[17]). The 3 parameterizations represent alternative iodide oxidation mechanisms, while iodate reduction in all 3 cases occurs quantitatively once local [O_2] is <10 µmol/kg (i.e., 'threshold'). Iodide oxidation parameterizations include: **Classic:** Iodide oxidation according to 'lifetime', where oxidation to iodate occurs ubiquitously according to first-order kinetics, regardless of ambient [O_2]. **ReMinO2LT:** Iodide oxidation according to 'reminO2lifetime', where oxidation to iodate occurs during O_2 consumption during organic remineralization, similar to ammonia oxidation during organic remineralization in cGENIE[18]. **Fennel:** Iodide oxidation according to 'Fennel', where oxidation to iodate occurs as a function of ambient [O_2] following Michaelis-Menten kinetics, analogous to ammonia reoxidation in cGENIE[19].

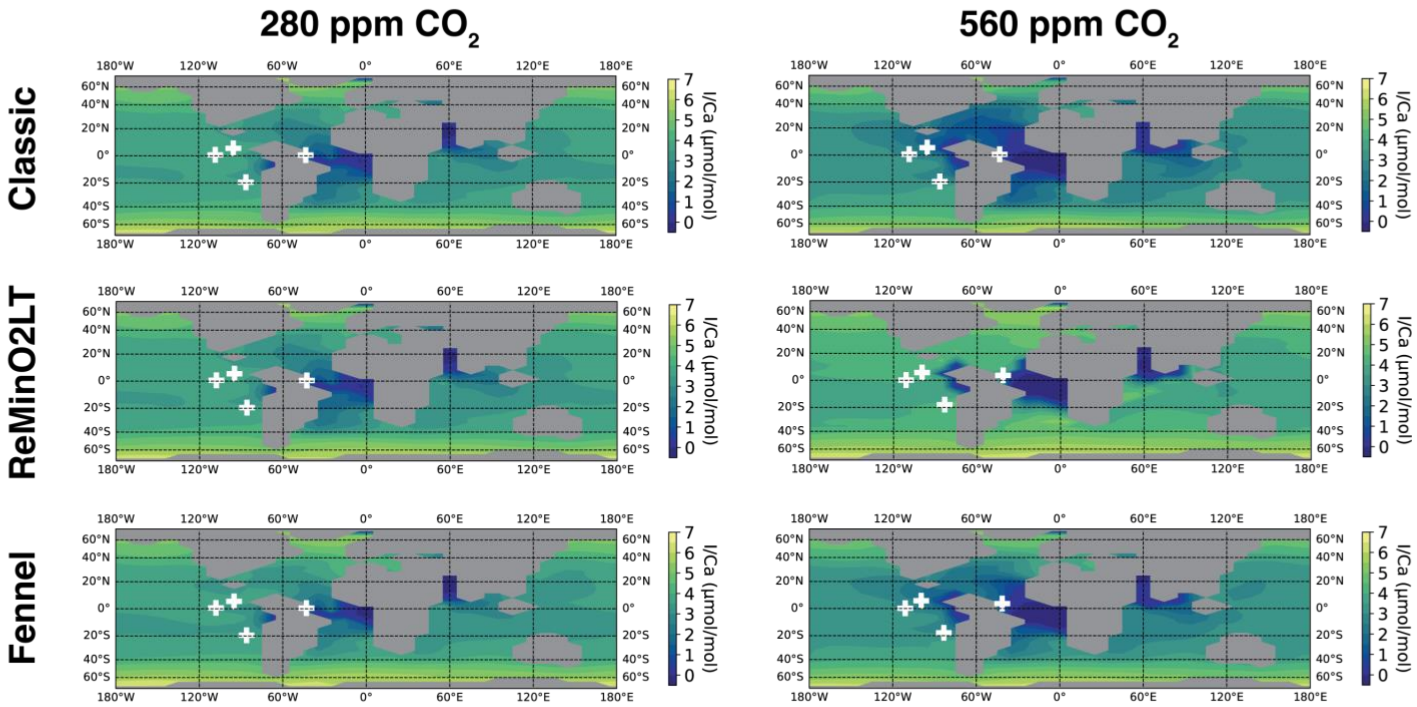

Supplementary Figure 7. Plots of I/Ca at 280 (first column) and 560 ppm (second column) CO_2 for each of the iodide oxidation schemes described in the previous Figure S6. The I/Ca is calculated from the iodate and temperature at the depth of minimum O_2. The partition coefficient for iodate incorporation into inorganic calcite has been demonstrated previously[20] to have a temperature-dependent relationship according to $K_D = 0.16\times T+13.65$. This temperature relationship is applied in the modeled I/Ca.

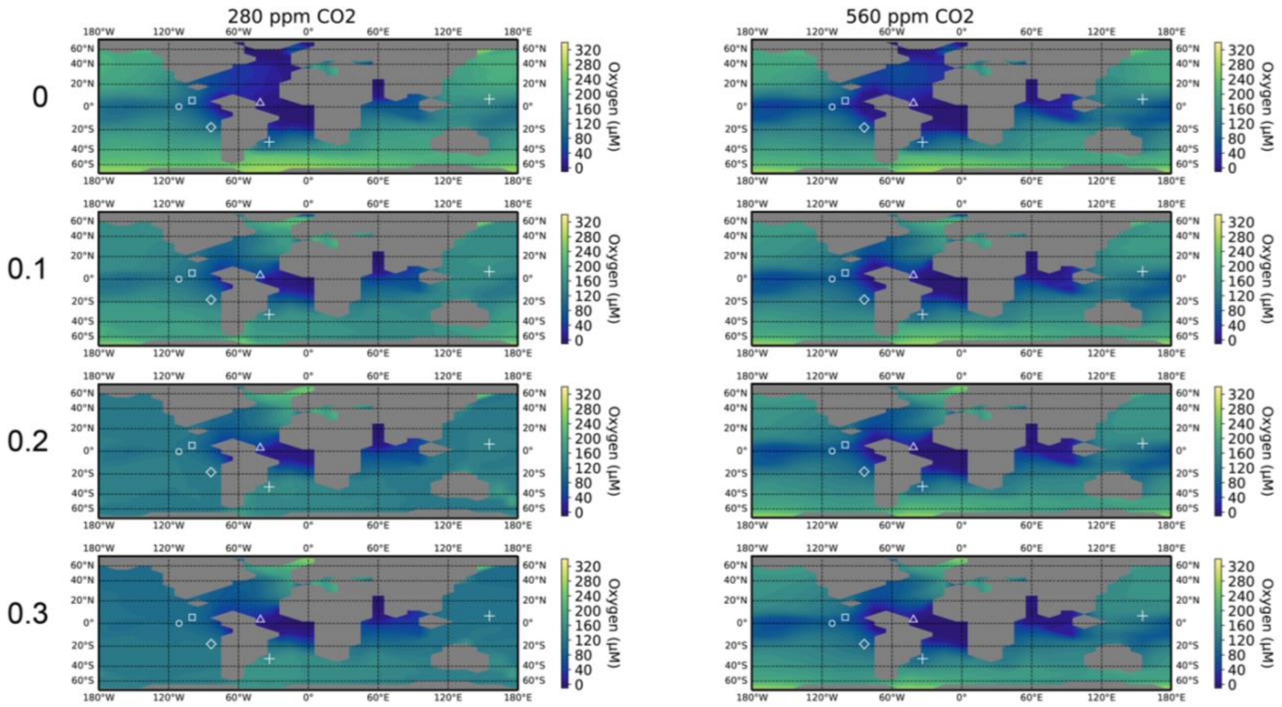

Supplementary Figure 8. Model oxygen in sensitivity analyses of varying salinity flux adjustments in North Atlantic. Miocene (15 Ma) minimum O_2 field simulated by cGENIE under low CO_2 (280 ppm, left panels) and high CO_2 (560 ppm, right panels) as well as under varying salinity flux adjustments. The salinity flux adjustments are shown on the left-hand side and are in sverdrups. The graphs demonstrate that under the high CO_2 Miocene scenario used in the main text, that the salinity flux adjustment has little impact on the oxygen deficient zone distributions unless the flux is eliminated, which limits downwelling as part of the Atlantic Meridional Overturning Current. In the main text Figures 2 and 3, a salinity flux adjustment of 0.1 Sv is used.

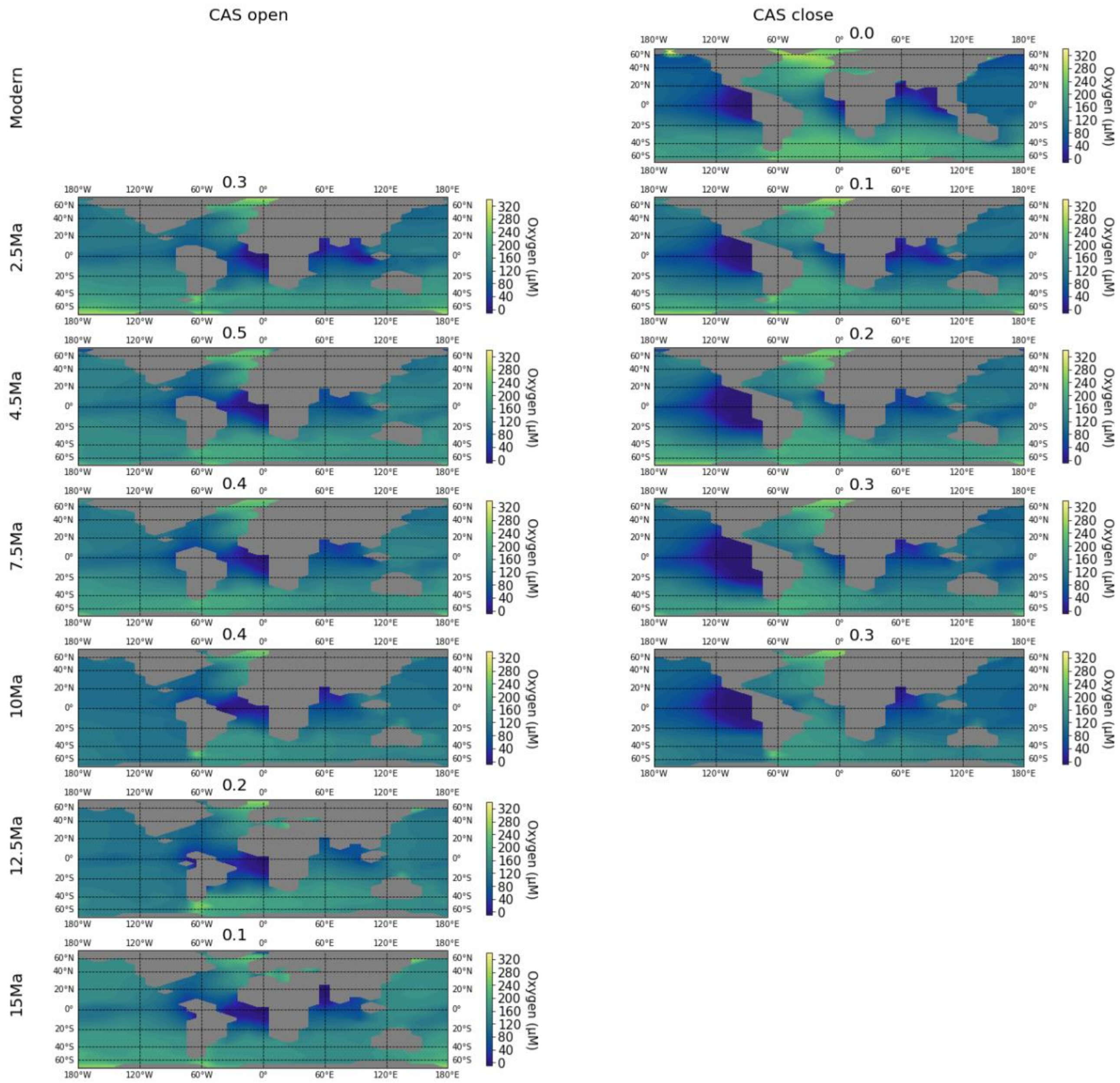

Supplementary Figure 9. Model sensitivity analyses of bathymetry on continental configuration impacts on marine oxygen (280 ppm CO_2). Minimum dissolved O_2 field simulated by cGENIE under a gradual bathymetry change from 15 Ma to the present. Simulations from 10 Ma to 2.5 Ma assess the effects of the Central American Seaway (CAS) being both open and closed on ocean currents. All paleo-bathymetry and 'salinity flux adjustments' settings follow the configurations of Crichton et al.[21], while the modern bathymetry uses Cheng et al.[22]. The salinity flux adjustments, in sverdrups, are shown above each figure. The results demonstrate that the opening and closing of the CAS has the biggest impact on Atlantic vs Pacific ODZ distribution.

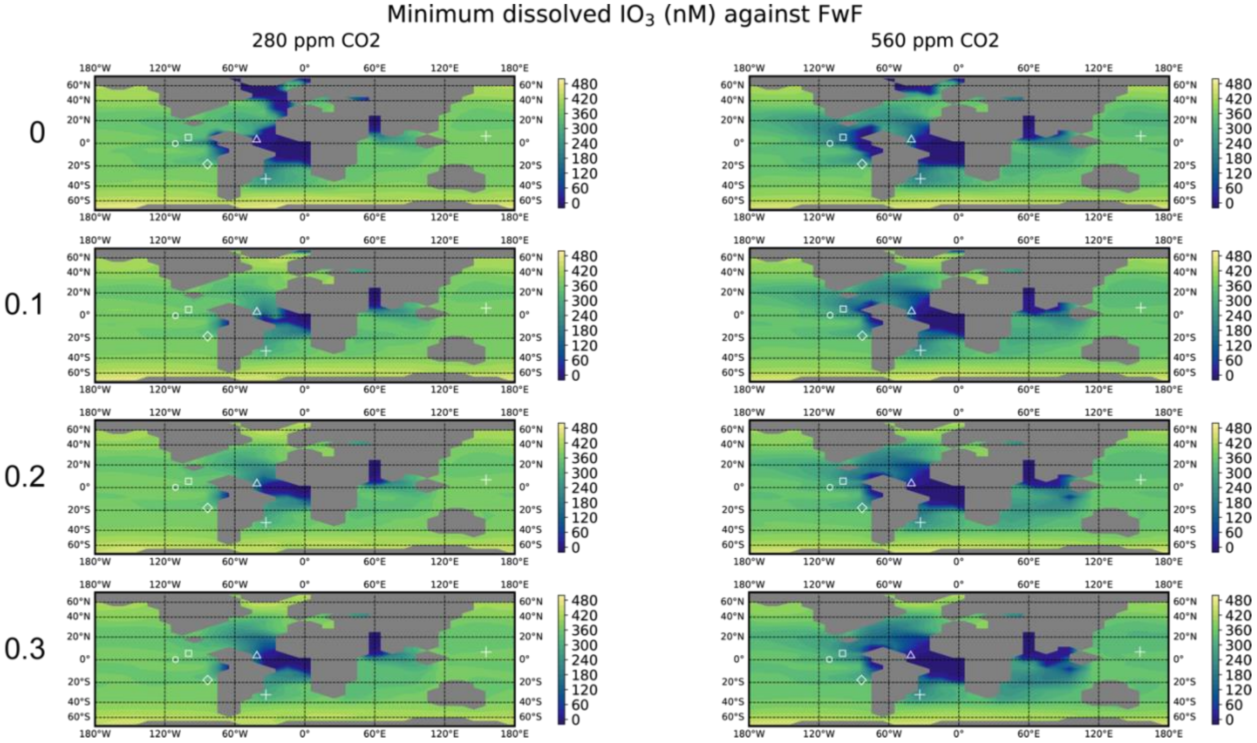

Supplementary Figure 10. Model iodate in sensitivity analyses of varying salinity flux adjustments (0, 0.1, 0.2, 0.3) in North Atlantic. Miocene (15Ma) minimum iodate field simulated by cGENIE under low CO_2 (280 ppm, left panels) and high CO_2 (560ppm, right panels) as well as under varying salinity flux adjustments. The 'fennel' iodate parameterization is used for these simulations. The salinity flux adjustments are shown on the left-hand side and are in sverdrups (Sv). The graphs demonstrate that under the high CO_2 Miocene scenario used in the main text, that the salinity flux adjustment has little impact on the the iodate distributions unless the flux is eliminated, which limits downwelling as part of the Atlantic Meridional Overturning Current. In the main text Figures 2 and 3, a salinity flux adjustment of 0.1 Sv is used.

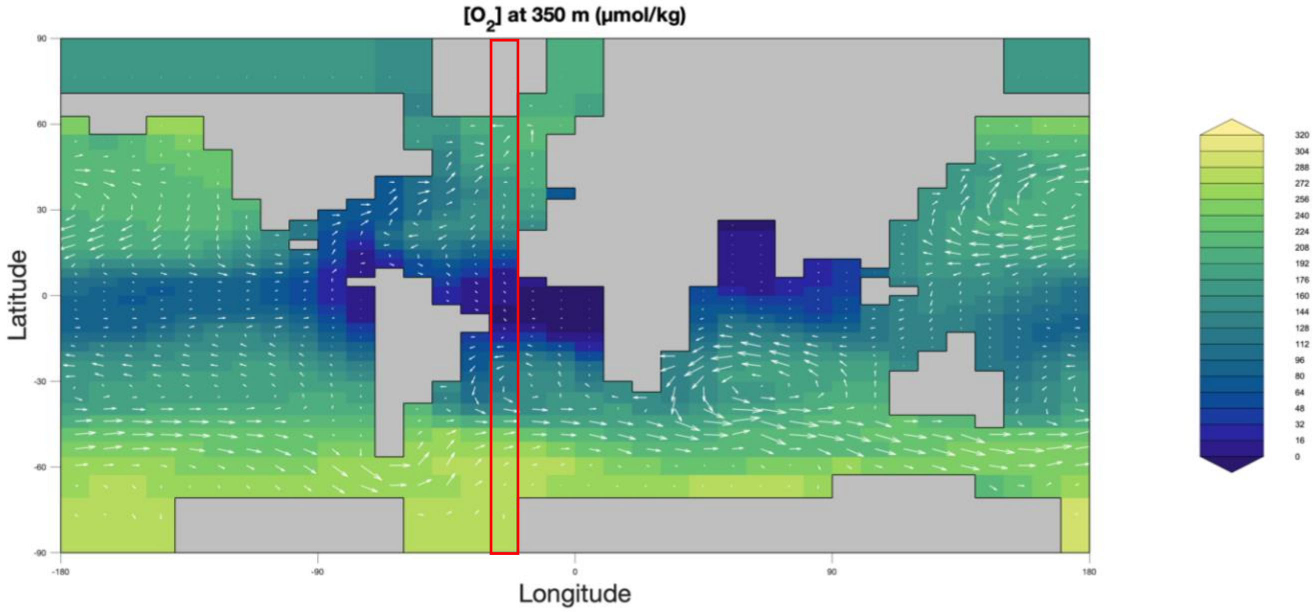

Supplementary Figure 11. Modeled oxygen distribution and current vectors at 350 m under Miocene conditions. The Miocene are for high CO_2 and Miocene continental configurations as shown in Figures 2 and 3 of the main text. The red box marks the grid cells for the meridional transect at 25° W shown in Supplementary Figures 13 and 14. Note that main text Figures 2 and 3 show minimum oxygen while this figure shows oxygen from a specific depth, determined to host the lowest O_2 water masses generally. Also shown here are vectors at this depth for water mass transport, with the size of the arrows scaled with the velocity. Low oxygen waters from the Pacific are transported through the Central American Seaway to the Atlantic, where they contribute to oxygen deficient zone formation in the mid-Atlantic.

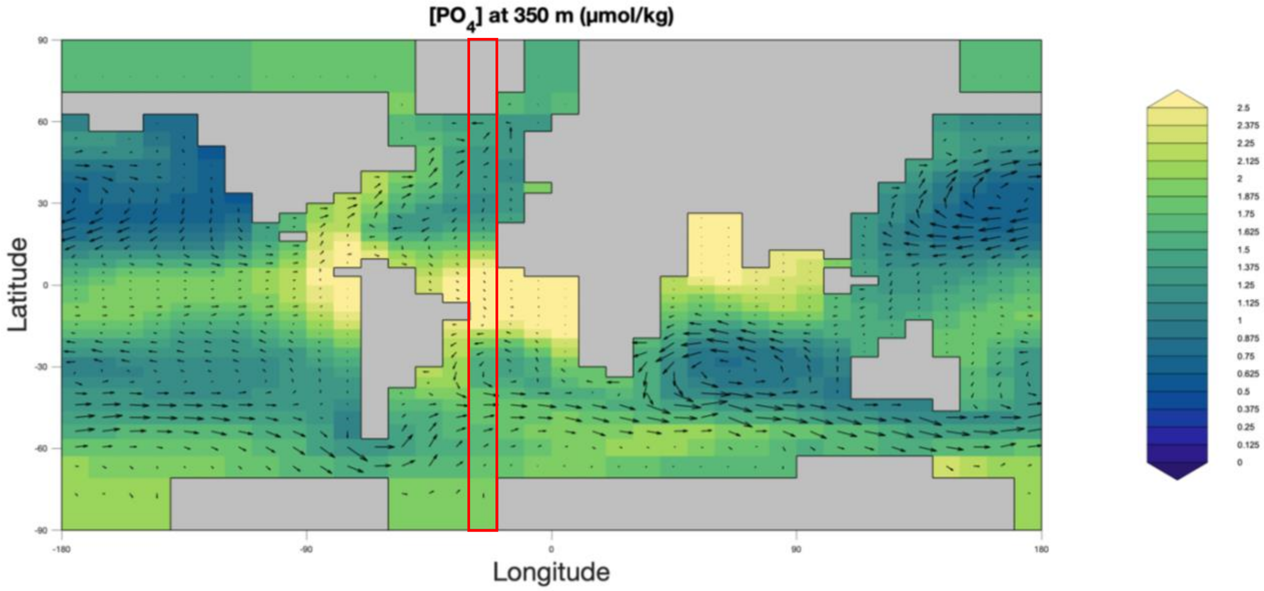

Supplementary Figure 12. Modeled phosphate distribution and current vectors at 350 m under Miocene conditions. Phosphate distribution at 350 m for the same model simulations shown for high CO_2 and Miocene continental configurations shown for oxygen in Figures 2 and 3 of the main text. The red box marks the grid cells for the meridional transect at 25° W shown in Supplementary Figures 13 and 14. The same vectors are shown here for water mass transport at this depth, with the size of the arrows scaled with the velocity. Waters from the Pacific are transported through the Central American Seaway to the Atlantic, where they accumulate nutrients (like phosphate) and contribute to oxygen deficient zone formation in the mid-Atlantic.

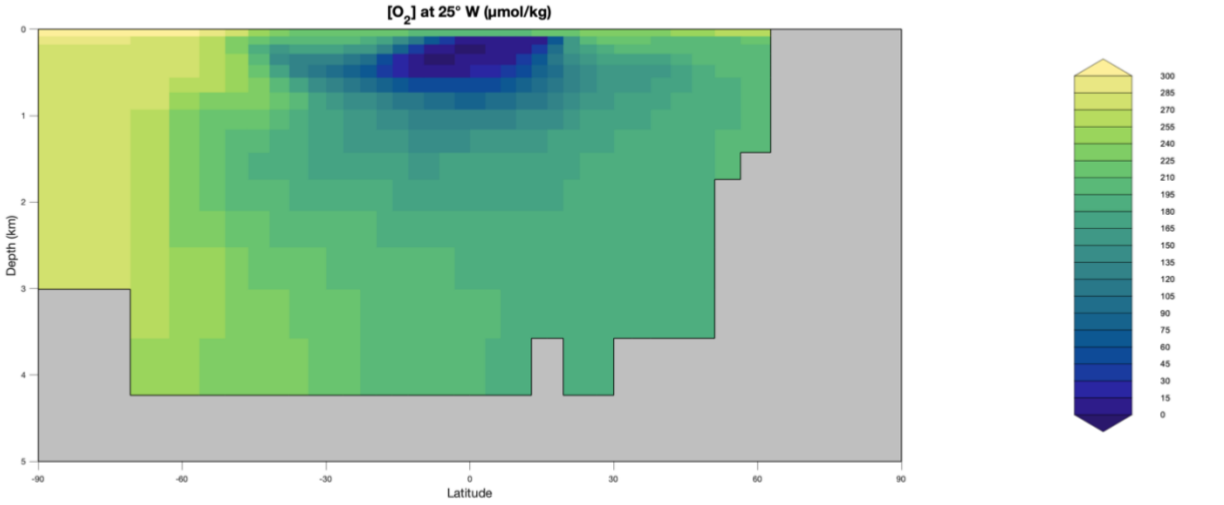

Supplementary Figure 13. North-South Atlantic transect of oxygen distribution. The Atlantic meridional transect of oxygen concentration is from the red box shown in Supplementary Figures 11 and 12.

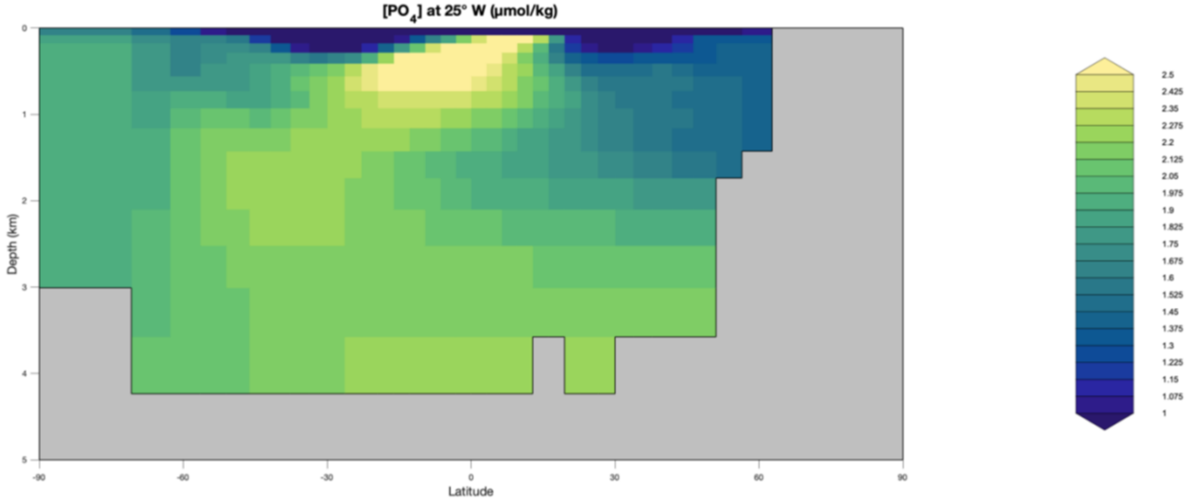

Supplementary Figure 14. North-South Atlantic transect of phosphate distribution. The Atlantic meridional transect of phosphate concentration is from the red box shown in Supplementary Figures 11 and 12.

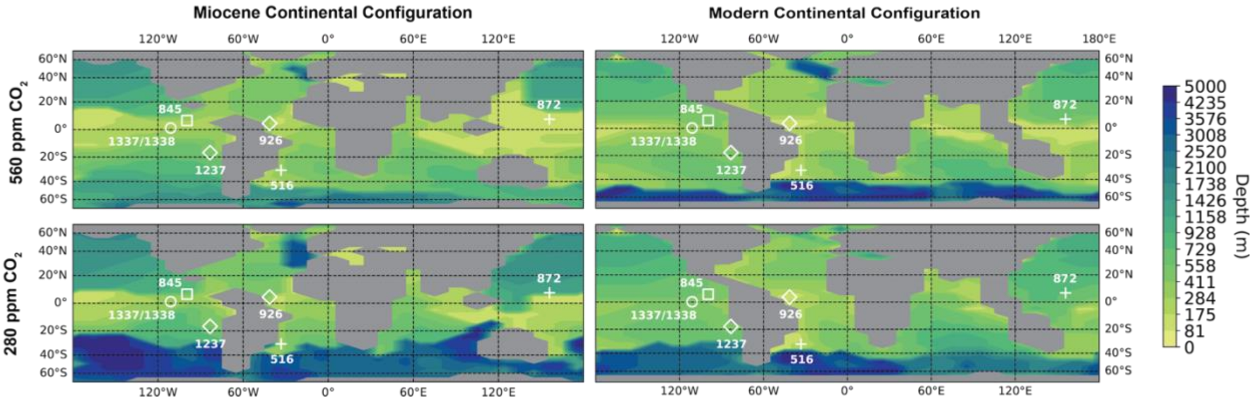

Supplementary Figure 15. Corresponding depths of minimum oxygen and associated iodate (or I/Ca) at 560 ppm CO_2 (top row) and 280 ppm CO_2 (bottom row) from Main Text Figure 2, Main Text Figure 3, Supplementary Figure 6, and Supplementary Figure 7.

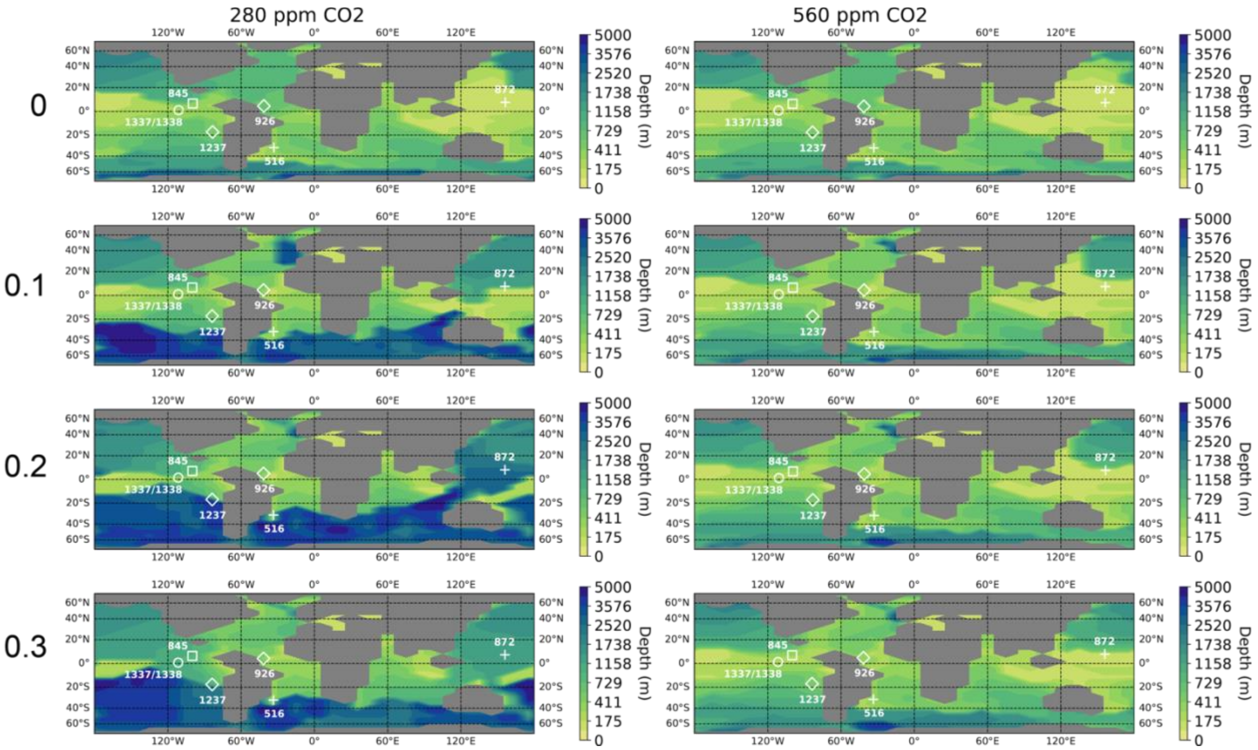

Supplementary Figure 16. Corresponding depths of minimum oxygen and corresponding iodate (or I/Ca) at variable salinity flux adjustments (0, 0.1, 0.2, 0.3, top to bottom) and CO_2 (280 ppm and 560 ppm left to right) from Supplementary Figure 8 and Supplementary Figure 10.

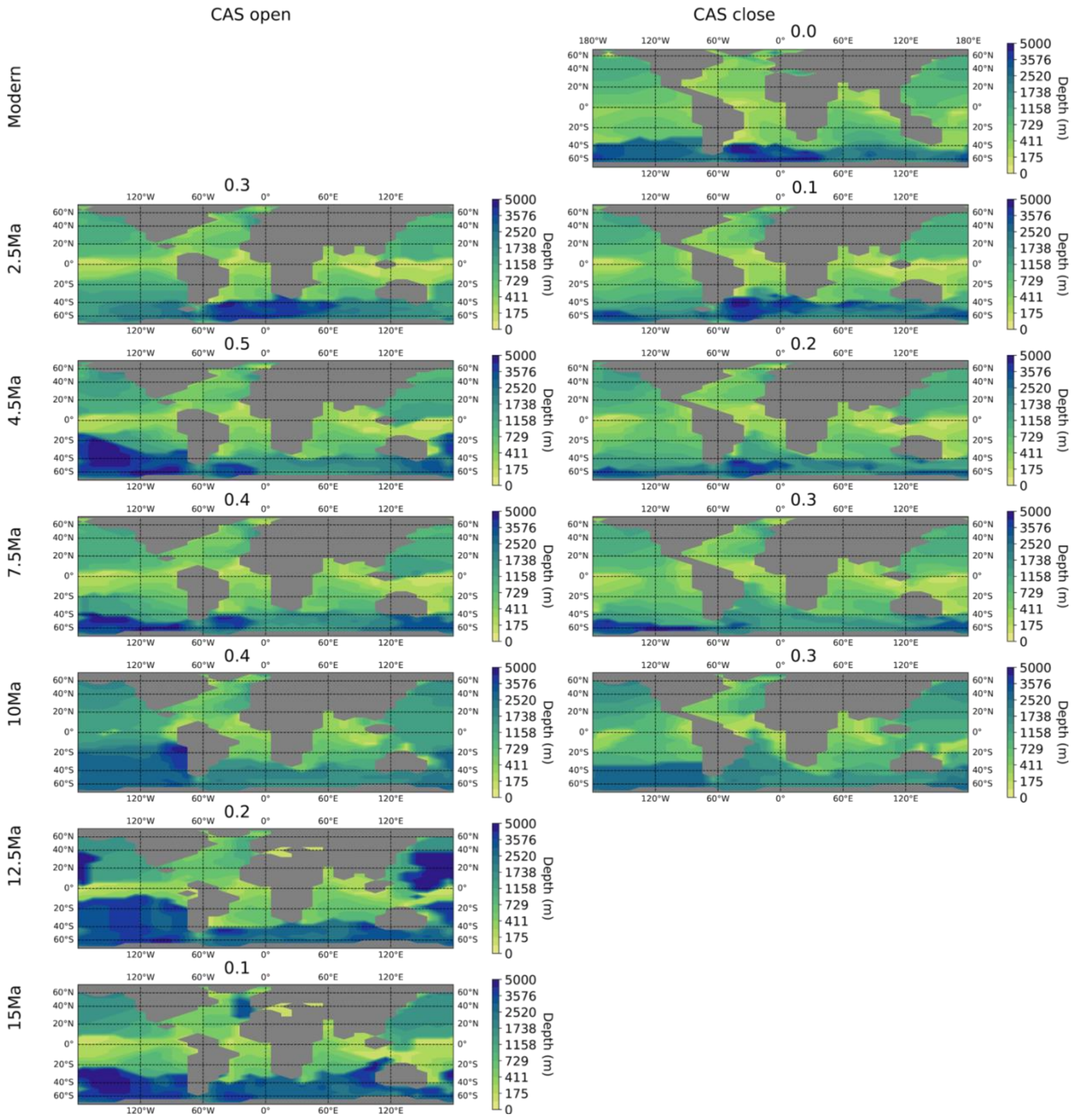

Supplementary Figure 17. Corresponding depths of minimum oxygen from Supplementary Figure 9. Left panel is for CAS open and right for CAS closed. Rows descend from youngest to oldest interval from top to bottom.

Supplementary Table 1. I/Ca Data: Sample information and I/Ca data for all new measurements used in this study. Sources for age estimates can be found in the "Sample Age Paleolocation Determination" section following the main text. Samples from ODP Leg 154 Site 926 where the presence of *Globorotaloides hexagonus* was observed are marked with an asterisk symbol (*), but note that not all samples were closely evaluated to document *Globorotaloides hexagonus.*

Exp.	Loc.	Hole	Core	Sec.	Sample Top (cm)	Sample Bottom (cm)	MCD	Age Estimate	Species	Run Date	I/Ca
154	926	B	32X	2W	75	77	320.06	15621.69	D. venezuelana	2/29/24	0.995
154	926	B	32X	2W	75	77	320.06	15621.69	D. venezuelana	2/29/24	1.844
154	926	B	32X	3W	65	67	321.46	15716.78	D. altispira	2/29/24	0.953
154	926	B	32X	3W	65	67	321.46	15716.78	D. altispira	2/29/24	0.939
*154	926	B	32X	4W	15	17	322.46	15784.69	D. altispira	7/11/23	1.748
*154	926	B	32X	4W	15	17	322.46	15784.69	D. venezuelana	7/11/23	1.296
*154	926	B	32X	4W	15	17	322.46	15784.69	T. sacculifer	7/11/23	2.041
154	926	B	32X	5W	13	15	323.94	15885.21	D. altispira	2/29/24	0.998
154	926	B	32X	5W	13	15	323.94	15885.21	D. venezuelana	2/29/24	1.121
154	926	B	32X	5W	13	15	323.94	15885.21	D. venezuelana	2/29/24	1.145
154	926	B	32X	5W	13	15	323.94	15885.21	D. venezuelana	4/24/23	1.005
154	926	B	32X	5W	114	116	319.66	15594.53	D. venezuelana	2/29/24	1.176
*154	926	B	32X	7W	13	15	326.94	16088.96	D. venezuelana	2/29/24	0.819
*154	926	B	32X	7W	13	15	326.94	16088.96	D. venezuelana	2/29/24	0.380
*154	926	B	33X	1W	16	18	327.57	16131.74	T. sacculifer	7/11/23	1.382
*154	926	B	33X	1W	16	18	327.57	16131.74	T. sacculifer	7/11/23	1.380
154	926	B	33X	1W	100	102	328.41	16188.79	T. trilobus	4/24/23	0.480
154	926	B	33X	1W	100	102	328.41	16188.79	T. trilobus	4/24/23	0.541
154	926	B	33X	1W	100	102	328.41	16188.79	D. altispira	6/14/23	0.909
154	926	B	33X	1W	100	102	328.41	16188.79	T. sacculifer	6/14/23	1.148

154	926	B	33X	2W	6	8	328.97	16226.83	T. sacculifer	6/14/23	0.957
154	926	B	33X	2W	6	8	328.97	16226.83	D. venezuelana	7/11/23	2.051
154	926	B	33X	2W	6	8	328.97	16226.83	D. altispira	7/11/23	1.051
154	926	B	33X	2W	6	8	328.97	16226.83	T. trilobus	7/11/23	1.035
154	926	B	33X	2W	6	8	328.97	16226.83	T. trilobus	7/11/23	1.019
154	926	B	33X	2W	54	56	329.45	16259.43	T. trilobus	7/11/23	0.922
154	926	B	33X	2W	54	56	329.45	16259.43	D. venezuelana	7/11/23	1.500
154	926	B	33X	2W	54	56	329.45	16259.43	T. sacculifer	7/11/23	1.670
*154	926	B	33X	2W	103	105	329.94	16292.71	T. trilobus	4/24/23	0.463
*154	926	B	33X	2W	103	105	329.94	16292.71	T. trilobus	4/24/23	0.902
*154	926	B	33X	2W	103	105	329.94	16292.71	D. altispira	6/14/23	1.014
*154	926	B	33X	2W	103	105	329.94	16292.71	T. sacculifer	6/14/23	0.959
*154	926	B	33X	2W	103	105	329.94	16292.71	D. altispira	2/29/24	0.614
*154	926	B	33X	2W	103	105	329.94	16292.71	D. venezuelana	2/29/24	0.328
*154	926	B	33X	3W	5	7	330.46	16328.02	T. trilobus	7/11/23	0.884
*154	926	B	33X	3W	5	7	330.46	16328.02	D. venezuelana	7/11/23	1.120
*154	926	B	33X	3W	5	7	330.46	16328.02	T. trilobus	7/11/23	1.132
*154	926	B	33X	3W	55	57	330.46	16328.02	T. sacculifer	4/24/23	1.189
*154	926	B	33X	3W	55	57	330.46	16328.02	T. sacculifer	2/29/24	1.005
154	926	B	33X	3W	105	107	331.46	16395.94	T. trilobus	4/24/23	1.046
154	926	B	33X	3W	105	107	331.46	16395.94	D. altispira	4/24/23	1.206
154	926	B	33X	3W	105	107	331.46	16395.94	T. trilobus	4/24/23	1.380
154	926	B	33X	3W	105	107	331.46	16395.94	D. altispira	4/24/23	1.253
*154	926	B	33X	6W	5	7	334.96	16633.64	T. trilobus	4/24/23	0.884
*154	926	B	33X	6W	5	7	334.96	16633.64	T. trilobus	4/24/23	0.868

*154	926	B	33X	6W	5	7	334.96	16633.64	T. trilobus	7/11/23	0.931
*154	926	B	33X	6W	5	7	334.96	16633.64	T. trilobus	7/11/23	0.945
*154	926	B	33X	6W	124	126	336.15	16714.46	D. altispira	2/29/24	1.182
*154	926	B	33X	6W	124	126	336.15	16714.46	D. venezuelana	2/29/24	1.009
*154	926	B	33X	6W	124	126	336.15	16714.46	D. altispira	2/29/24	0.918
154	926	B	34X	2W	35	37	338.96	16905.31	D. altispira	2/29/24	0.616
*154	926	C	32X	5W	75	77	326.06	16029.19	G. dehiscens	7/11/23	1.592
*154	926	C	32X	5W	75	77	326.06	16029.19	T. sacculifer	7/11/23	1.828
202	1237	B	22H	4W	132	134	221.69	14699.55	D. venezuelana	3/18/21	6.771
202	1237	B	22H	4W	132	134	221.69	14699.55	G. dehiscens	3/18/21	3.026
202	1237	B	22H	5W	16	18	222.04	14777.50	D. venezuelana	6/10/21	3.200
202	1237	B	22H	5W	16	18	222.04	14887.92	D. venezuelana	6/10/21	3.577
202	1237	B	22H	5W	16	18	222.04	14887.92	G. dehiscens	6/10/21	2.122
202	1237	B	22H	5W	131	133	222.87	14932.63	Dentiglobigerina spp.	10/18/21	4.273
202	1237	B	22H	5W	131	133	222.87	14932.63	G.dehiscens	10/18/21	2.783
202	1237	B	22H	5W	131	133	222.87	14932.63	Dentiglobigerina spp.	10/18/21	4.118
202	1237	B	22H	6W	15	17	223.55	15058.30	D. venezuelana	6/10/21	5.447
202	1237	B	22H	6W	15	17	223.55	15058.30	G.dehiscens	6/10/21	4.340
202	1237	B	22H	6W	15	17	223.55	15058.30	D. venezuelana	6/10/21	4.905
202	1237	B	23H	3W	126	128	231.43	16305.60	D. venezuelana	6/10/21	3.253
202	1237	B	23H	3W	126	128	231.43	16305.60	G.dehiscens	6/10/21	2.120
202	1237	B	23H	4W	16	18	231.84	16396.80	T. trilobus	3/18/21	1.331
202	1237	B	23H	4W	16	18	231.84	16396.80	G.dehiscens	3/18/21	1.754
202	1237	B	23H	4W	16	18	231.84	16396.80	D. venezuelana	3/18/21	3.870

202	1237	B	23H	4W	16	18	231.84	16396.80	T. trilobus	3/18/21	1.111
202	1237	B	23H	5W	36	38	233.54	16798.00	G.dehiscens	6/10/21	2.111
202	1237	C	22H	2W	51	53	224.22	15215.13	D. venezuelana	6/10/21	3.578
202	1237	C	22H	2W	51	53	224.22	15215.13	D. venezuelana	6/10/21	3.064
202	1237	C	22H	2W	51	53	224.22	15215.13	D. altispira	6/10/21	3.234
202	1237	C	22H	2W	136	138	225.07	15309.01	T. trilobus	10/18/21	0.869
202	1237	C	22H	2W	136	138	225.07	15309.01	D. venezuelana	10/18/21	0.928
202	1237	C	22H	4W	41	43	227.14	15618.27	D. venezuelana	6/10/21	3.980
202	1237	C	22H	4W	41	43	227.14	15618.27	G.dehiscens	6/10/21	1.849
202	1237	C	22H	4W	41	43	227.14	15618.27	T. trilobus	6/10/21	0.517
202	1237	C	22H	4W	96	98	227.66	15697.36	D. spp	10/18/21	3.428
202	1237	C	22H	4W	96	98	227.66	15697.36	G.dehiscens	10/18/21	3.230
202	1237	C	22H	4W	96	98	227.66	15697.36	D. venezuelana	11/13/21	3.294
202	1237	C	22H	4W	131	133	228.16	15758.21	D. venezuelana	10/18/21	3.611
202	1237	C	22H	4W	131	133	228.16	15758.21	G.dehiscens	10/18/21	2.715
202	1237	C	22H	5W	82	84	229.06	15890.87	G.dehiscens	3/18/21	3.887
202	1237	C	22H	5W	82	84	229.06	15890.87	D. venezuelana	3/18/21	5.340
202	1237	C	22H	5W	116	118	229.40	15939.89	D. venezuelana	10/18/21	3.981
202	1237	C	22H	5W	116	118	229.40	15939.89	G.dehiscens	10/18/21	2.885
202	1237	C	22H	5W	116	118	229.40	15939.89	D. altispira	10/18/21	4.423
202	1237	C	22H	5W	116	118	229.40	15939.89	G.dehiscens	11/13/21	2.366
202	1237	C	22H	5W	116	118	229.40	15939.89	G.dehiscens	11/13/21	2.256
202	1237	C	23H	3W	6	8	235.48	17294.20	D. venezuelana	3/18/21	5.911
202	1237	C	23H	3W	6	8	235.48	17294.20	D. altispira	3/18/21	5.297
202	1237	C	23H	3W	6	8	235.48	17294.20	D. venezuelana	10/18/21	4.211

321	U1337	A	34X	3W	27	29	341.52	15285.41	D. venezuelana	10/18/21	0.669
321	U1337	A	35X	3W	34	36	350.91	15602.30	D. venezuelana	3/18/21	0.753
321	U1337	A	35X	3W	34	36	350.91	15602.30	D. venezuelana	3/18/21	1.009
321	U1337	A	36X	4W	133	135	364.52	16274.90	D. venezuelana	3/18/21	0.533
321	U1337	A	36X	4W	133	135	364.52	16274.90	D. venezuelana	3/18/21	0.264
321	U1337	C	18X	5W	106	108	340.20	15205.25	D. venezuelana	3/18/21	0.972
321	U1337	C	18X	5W	106	108	340.20	15205.25	D. venezuelana	10/18/21	0.868
321	U1337	C	20X	4W	74	76	359.60	16045.33	D. venezuelana	3/18/21	1.007
321	U1337	C	20X	4W	74	76	359.60	16045.33	D. venezuelana	3/18/21	0.759
321	U1337	C	20X	4W	74	76	359.60	16045.33	D. venezuelana	11/13/21	0.808
321	U1337	C	20X	5W	74	76	359.60	16045.33	D. venezuelana	11/13/21	0.707
321	U1337	D	36X	3W	94	96	352.43	15680.49	D. venezuelana	3/18/21	0.820
321	U1337	D	36X	3W	94	96	352.43	15680.49	D. venezuelana	3/18/21	0.671
321	U1337	D	36X	4W	140	142	354.39	15799.05	D. venezuelana	3/18/21	1.039
321	U1337	D	36X	4W	140	142	354.39	15799.05	G.dehiscens	3/18/21	0.582
321	U1338	B	41H	1W	102	104	413.09	15178.14	D. venezuelana	10/16/21	0.635
321	U1338	B	41H	1W	137	139	413.44	15189.60	D. venezuelana	6/10/21	0.928
321	U1338	B	41H	3W	102	104	416.09	15323.87	D. spp	6/10/21	0.195
321	U1338	B	41H	3W	102	104	416.09	15323.87	G.dehiscens	6/10/21	0.000
321	U1338	B	41H	3W	102	104	416.09	15323.87	D. altispira	6/10/21	0.000
321	U1338	B	41H	3W	102	104	416.09	15323.87	G. subquadratus	6/10/21	0.000
321	U1338	B	42H	2W	122	124	424.66	15568.65	D. venezuelana	6/10/21	0.557
321	U1338	B	42H	2W	122	124	424.66	15568.65	D. venezuelana	6/10/21	0.280
321	U1338	B	42H	2W	122	124	424.66	15568.65	G.dehiscens	6/10/21	0.082
321	U1338	B	42H	2W	122	124	424.66	15568.65	D. venezuelana	6/10/21	0.174

321	U1338	B	42H	3W	138	140	426.34	15637.03	D. venezuelana	11/13/21	0.892
321	U1338	B	42H	3W	138	140	426.34	15637.03	D. venezuelana	11/13/21	0.921
321	U1338	C	40H	1W	93	95	393.01	14522.76	D. venezuelana	6/10/21	0.400
321	U1338	C	40H	1W	93	95	393.01	14522.76	D. venezuelana	6/10/21	0.530
321	U1338	C	40H	4W	42	44	397.02	14639.20	D. venezuelana	6/10/21	0.101
321	U1338	C	40H	4W	42	44	397.02	14639.20	T. trilobus	6/10/21	0.000
321	U1338	C	43H	2W	82	84	420.67	15476.92	D. venezuelana	10/16/21	0.726

Supplementary Table 2. JCP-1 I/Ca: I/Ca values for all ICP-MS runs used to generate new data for this study.

ICP-MS Run Date	JCP-1 I/Ca μmol/mol
**11/13/20**	3.71
**12/12/20**	3.75
**1/28/21**	3.96
**3/18/21**	3.84
**6/10/21**	4.24
**10/18/21**	3.82
**11/16/21**	3.71
**4/24/23**	4.12
**5/9/23**	4.02
**5/9/23**	4.02
**6/14/23**	4.03
**7/11/23**	4.26
**2/29/24**	4.05

Supplementary Table 3. Preservation Assessment: Summary of SEM analysis.

Sample	Specimen Code	View	Preservation Rating
1237B-23H-4W-16/18	A-9-1a	external closeup	poor
1237B-23H-4W-16/18	A-9-1b	whole specimen external	fair/poor
1237B-23H-4W-16/18	A-9-2a	cross-section	poor
1237B-23H-4W-16/18	A-9-2b	internal wall close-up	poor
1237B-23H-4W-16/18	A-9-3a	external closeup	poor
1237B-23H-4W-16/18	A-9-4a	internal wall close-up	poor
1237B-23H-4W-16/18	A-10-1a	external closeup	poor
1237B-23H-4W-16/18	A-10-1b	whole specimen external	poor
1237B-23H-4W-16/18	A-10-2	external closeup	poor
1237C-22H-2W-51	B-1-1	cross	moderate
1237C-22H-2W-51	B-1-1b	internal wall close-up	moderate/poor
1237C-22H-2W-51	B-1-2	external closeup	poor
1237C-22H-2W-51	B-2-1	Cross-section	poor
1237C-22H-2W-51	B-2-1b	internal wall close-up	poor
1237C-22H-2W-51	B-2-2	external closeup	moderate/poor
1237C-22H-2W-51	B-2-3	external closeup	moderate/poor
1237C-22H-2W-51	B-3-1	cross section and internal	poor
1237C-22H-2W-51	B-3-1b	internal wall close-up	poor
1237C-22H-2W-51	B-3-2	external closeup	moderate/poor
1237C-22H-4W-96	A-11-1a	cross-section	poor
1237C-22H-4W-96	A-12-1a	external closeup	poor
1237C-22H-4W-96	A-12-1b	whole specimen external	poor
1237C-22H-4W-96	A-12-2a	cross-section	poor
1337A-35X-3W-8/10	A-4-1a	external closeup	fair/poor
1337A-35X-3W-8/10	A-4-1b	whole specimen external	fair
1337A-35X-3W-8/10	A-4-2a	cross-section	good/moderate
1337A-35X-3W-8/10	A-4-2b	internal wall close-up	moderate/fair
1337A-35X-3W-8/10	A-4-5-1a	external closeup	moderate/fair
1337A-35X-3W-8/10	A-5-1b	whole specimen external	good/moderate
1337A-35X-3W-8/10	A-5-2a	cross-section	moderate
1337A-35X-3W-8/10	A-5-2b	internal wall close-up	fair
1337C-18X-5W-106/108	A-6-1a	whole specimen external	good/moderate
1337C-18X-5W-106/108	A-6-2a	internal wall close-up	fair/poor

1337C-18X-5W-106/108	A-6-2b	cross-section	poor
1337C-18X-5W-106/108	A-7-1a	external closeup	poor
1337C-18X-5W-106/108	A-7-1b	whole specimen external	fair/poor
1337C-18X-5W-106/108	A-7-2a	cross-section	poor
1337C-18X-5W-106/108	A-7-2b	internal wall close-up	poor
1337C-18X-5W-106/108	A-7-3a	cross-section	poor
1337C-18X-5W-106/108	A-8-1	whole specimen external	fair
1337C-20X-5W-74	A-1	Large broken specimen	good/very good
1337C-20X-5W-74	A-1-2	internal wall close-up	good/very good
1337C-20X-5W-74	A-1	cross-section close-up	good/very good
1337C-20X-5W-74	A-2-1	whole specimen external	fair/good
1337C-20X-5W-74	A-2-2	whole specimen external	fair
1337C-20X-5W-74	A-2-2	external closeup	fair/poor
1337C-20X-5W-74	A-3-1a	cross-section/internal wall	fair
1337C-20X-5W-74	A-3-1b	Internal wall close-up	good/fair
1337C-20X-5W-74	A-3-1c	external closeup	fair
1337C-20X-5W-74	A-3-2	whole specimen external	fair/poor
1338-40H-6W-22	B-4-1	cross	good/moderate
1338-40H-6W-22	B-4-1	internal wall close-up	good/moderate
1338-40H-6W-22	B-4-1c	external closeup	moderate/poor
1338-40H-6W-22	B-6-1	external closeup	moderate/poor
1338-40H-6W-22	B-6-2	external closeup	moderate
1338-40H-6W-22	B-7-1	external closeup	moderate/poor
1338-40H-6W-22	B-7-2	cross	good/moderate
1338-40H-6W-22	B-7-2b	internal wall close-up	moderate
1338-40H-6W-22	B-7-3	external closeup	moderate
1338B-41-1W-137	B-10-1a	cross-section	good
1338B-41-1W-137	B-10-2a	internal wall close-up	good/moderate
1338B-41-1W-137	B-11-1a	cross	moderate
1338B-41-1W-137	B-11-1b	internal wall close-up	moderate/poor
1338B-41-1W-137	B-11-2a	whole specimen external	moderate/poor
1338B-42-2-122	B-8-1	cross	moderate
1338B-42-2-122	B-8-1b	internal wall close-up	good/moderate
1338B-42-2-122	B-8-2	external closeup	good
1338B-42-2-122	B-8-2b	whole specimen external	good
1338B-42-2-122	B-8-3	external closeup	moderate/poor
1338B-42-2W-122	B-9-1a	cross-section	good
1338B-42-2W-122	B-9-2a	external closeup	moderate/ poor
1338C-40H-6W-22	B-4-2	external closeup	good/moderate
926B-32x-1W-15	C-5-1a	external closeup	moderate/poor
926B-32x-1W-15	C-6-1a	cross-section	moderate/poor
926B-32x-1W-15	C-6-1b	internal wall close-up	moderate/poor
926B-32x-1W-15	C-6-2a	whole specimen external	moderate/poor
926B-32x-1W-15	C-7-1a	internal wall close-up	moderate
926B-32x-1W-15	C-7-2a	cross-section	moderate
926B-32x-1W-15	C-7-3a	external closeup	moderate
926B-33X-1W-5	C-8-1a	external closeup	good/moderate
926B-33X-1W-5	C-8-2a	cross-section	good/moderate
926B-33X-1W-5	C-8-2a	internal wall close-up	moderate
926B-33X-1W-5	C-9-1a	cross	moderate/poor
926B-33X-1W-5	C-9-1a	external closeup	moderate
926B-33x-2w-103	C-1-1a	cross section	moderate
926B-33x-2w-103	C-1-2a	internal wall close-up	moderate/poor
926B-33x-2w-103	C-1-3a	external closeup	good/moderate
926B-33x-2w-103	C-2-1a	internal wall close-up	moderate
926B-33x-2w-103	C-2-2a	external closeup	moderate
926B-33x-2w-103	C-2-2b	whole specimen external	good/moderate
926B-33x-2w-103	C-3-1a	cross-section	good/moderate
926B-33x-2w-103	C-3-1b	external closeup	good/moderate
926B-33x-2w-103	C-4-1a	external closeup	moderate/poor

Supplementary Table 4. ODP Site 926 Ages: Age-Depth correlation from Sosdian et al., 2019 (supplement to Sosdian et al.[23]) used to constrain ages of ODP 154-926 samples used in this study.

Event	Sample comment	Site	Core	Sect	Depth Top	Depth Bottom	Depth (mcd)	Age [ka BP]
**154-926B**	T. trilobus (300-355 µm)	926B	31	2	98	99.5	310.6	14950
**154-926B**	T. trilobus (300-355 µm)	926B	31	3	18	19.5	311.3	15000
**154-926B**	T. trilobus (300-355 µm)	926B	31	7	8	9.5	317.2	15430
**154-926B**	T. trilobus (300-355 µm)	926B	32	3	78	79.5	321.6	15740
**154-926B**	T. trilobus (300-355 µm)	926B	32	5	2	3.5	323.8	15900
**154-926B**	T. trilobus (300-355 µm)	926B	32	6	30	32	325.6	16020
**154-926B**	T. trilobus (300-355 µm)	926B	32	6	38, 46, 50	40, 48, 52	325.8	16030
**154-926B**	T. trilobus (300-355 µm)	926B	32	6	110, 118	112, 120	326.5	16080
**154-926B**	T. trilobus (300-355 µm)	926B	34	1	139	140.5	338.5	16870
**154-926B**	T. trilobus (300-355 µm)	926B	34	2	45, 49	46.5, 50.5	339.1	16900
**154-926B**	T. trilobus (300-355 µm)	926B	34	CC	10.5, 18.5	12, 20	342.1	17090

Supplementary Table 5. List of cGENIE experiments

cGENIE experiment number	Iodine cycle parameters				CO_2 concentration (ppm)	Continental configuration	
	IO_3^- reduction threshold (μM O_2)	I- oxidation half-saturation constant (μM O_2)	Maximum I^- oxidation rate constant ($year^{-1}$)	I:C ratio ($\times 10^{-4}$ mol/mol)		Age (Ma)	CAS
1	10	20	0.1	3.5	280	0	Open
2	10	20	0.1	3.5	280	2.5	Open
3	10	20	0.1	3.5	280	2.5	Closed
4	10	20	0.1	3.5	280	4.5	Open
5	10	20	0.1	3.5	280	4.5	Closed
6	10	20	0.1	3.5	280	7.5	Open
7	10	20	0.1	3.5	280	7.5	Closed
8	10	20	0.1	3.5	280	10	Open
9	10	20	0.1	3.5	280	10	Closed
10	10	20	0.1	3.5	280	12.5	Open
11	10	20	0.1	3.5	280	15	Open
12	10	20	0.1	3.5	560	0	Open
13	10	20	0.1	3.5	560	2.5	Open
14	10	20	0.1	3.5	560	2.5	Closed
15	10	20	0.1	3.5	560	4.5	Open
16	10	20	0.1	3.5	560	4.5	Closed
17	10	20	0.1	3.5	560	7.5	Open
18	10	20	0.1	3.5	560	7.5	Closed
19	10	20	0.1	3.5	560	10	Open
20	10	20	0.1	3.5	560	10	Closed
21	10	20	0.1	3.5	560	12.5	Open
22	10	20	0.1	3.5	560	15	Open

Supplementary Table 6. Modern and paleo coordinates for study locations. Also includes modern water depths. Details for source of paleocoordinates provided in the "Sample Age and Paleolocation Determination" section of main text.

Site	Modern Latitude (°N)	Paleo-Latitude (°N)	Modern Longitude (°W)	Paleo-Longitude (°W)	Modern water depth (m)
IODP 320-U1337	2.3	0.2133	-117.58	-111.0398	4,464
ODP 202-1237	16.04	-17.43	-76.11	-83.29	3,212
ODP 138-845	9.34	5.608	-94.35	-99.543	3,704
ODP 154-926	3.7	3.575	-42.9	-41.344	3,610

References

1 Westerhold, T. *et al.* An astronomically dated record of Earth's climate and its predictability over the last 66 million years. *Science* **369**, 1383-1387 (2020).
2 Holbourn, A., Kuhnt, W., Kochhann, K. G. D., Andersen, N. & Meier, K. J. S. Global perturbation of the carbon cycle at the onset of the Miocene Climatic Optimum. *Geology* **43**, 123-126 (2015). https://doi.org/10.1130/g36317.1
3 Holbourn, A., Kuhnt, W., Schulz, M., Flores, J. A. & Andersen, N. Orbitally-paced climate evolution during the middle Miocene "Monterey" carbon-isotope excursion. *Earth and Planetary Science Letters* **261**, 534-550 (2007). https://doi.org/10.1016/j.epsl.2007.07.026
4 Hess, A. V. *et al.* A well-oxygenated eastern tropical Pacific during the warm Miocene. *Nature* **619**, 521-+ (2023). https://doi.org/10.1038/s41586-023-06104-6
5 Zhou, X., Hess, A. V., Bu, K., Sagawa, T. & Rosenthal, Y. Simultaneous determination of I/Ca and other elemental ratios in foraminifera: Comparing results from acidic and basic solutions. *Geochemistry, Geophysics, Geosystems* **23**, e2022GC010660 (2022).
6 Lu, W. Y. *et al.* I/Ca in epifaunal benthic foraminifera: A semi-quantitative proxy for bottom water oxygen in a multi-proxy compilation for glacial ocean deoxygenation. *Earth and Planetary Science Letters* **533** (2020). https://doi.org/ARTN 116055 10.1016/j.epsl.2019.116055
7 Lu, W. Y., Wang, Y., Oppo, D. W., Nielsen, S. G. & Costa, K. M. Comparing paleo-oxygenation proxies (benthic foraminiferal surface porosity, I/Ca, authigenic uranium) on modern sediments and the glacial Arabian Sea. *Geochimica Et Cosmochimica Acta* **331**, 69-85 (2022). https://doi.org/10.1016/j.gca.2022.06.001
8 Hess, A. V., Rosenthal, Y., Zhou, X. & Bu, K. The I/Ca paleo-oxygenation proxy in planktonic foraminifera: A multispecies core-top calibration. *Geochimica et Cosmochimica Acta* (2025).
9 Moriyasu, R. *et al.* Meridional survey of the central pacific reveals iodide accumulation in equatorial surface waters and benthic sources in the abyssal plain. *Global Biogeochemical Cycles* **37**, e2021GB007300 (2023).
10 Rapp, I. *et al.* El Niño-driven oxygenation impacts Peruvian shelf iron supply to the South Pacific Ocean. *Geophys Res Lett* **47**, e2019GL086631 (2020).
11 Cutter, G. A., Moffett, J. G., Nielsdóttir, M. C. & Sanial, V. Multiple oxidation state trace elements in suboxic waters off Peru: In situ redox processes and advective/diffusive horizontal transport. *Marine Chemistry* **201**, 77-89 (2018).
12 Moriyasu, R., Evans, N., Bolster, K. M., Hardisty, D. S. & Moffett, J. W. The Distribution and Redox Speciation of Iodine in the Eastern Tropical North Pacific Ocean. *Global Biogeochemical Cycles* **34** (2020). https://doi.org/ARTN e2019GB006302 10.1029/2019GB006302
13 Rue, E. L., Smith, G. J., Cutter, G. A. & Bruland, K. W. The response of trace element redox couples to suboxic conditions in the water column. *Deep Sea Research Part I: Oceanographic Research Papers* **44**, 113-134 (1997).
14 Rapp, I. *et al.* Controls on redox-sensitive trace metals in the Mauritanian oxygen minimum zone. *Biogeosciences* **16**, 4157-4182 (2019).

15 Chapman, P. CHANGES IN IODINE SPECIATION IN THE BENGUELA CURRENT UPWELLING SYSTEM. *Deep-Sea Research Part a-Oceanographic Research Papers* **30**, 1247-1259 (1983). https://doi.org/10.1016/0198-0149(83)90083-3
16 Schlitzer, R. *et al.* The GEOTRACES intermediate data product 2017. *Chemical Geology* **493**, 210-223 (2018).
17 Cheng, K., Ridgwell, A. & Hardisty, D. S. Characterizing the marine iodine cycle and its relationship to ocean deoxygenation in an Earth system model. *Biogeosciences* **21**, 4927-4949 (2024).
18 Martin, T. S., Primeau, F. & Casciotti, K. L. Modeling oceanic nitrate and nitrite concentrations and isotopes using a 3-D inverse N cycle model. *Biogeosciences* **16**, 347-367 (2019).
19 Fennel, K., Follows, M. & Falkowski, P. G. The co-evolution of the nitrogen, carbon and oxygen cycles in the Proterozoic ocean. *American Journal of Science* **305**, 526-545 (2005).
20 Zhou, X., Thomas, E., Rickaby, R. E., Winguth, A. M. & Lu, Z. I/Ca evidence for upper ocean deoxygenation during the PETM. *Paleoceanography* **29**, 964-975 (2014).
21 Crichton, K. A., Ridgwell, A., Lunt, D. J., Farnsworth, A. & Pearson, P. N. Data-constrained assessment of ocean circulation changes since the middle Miocene in an Earth system model. *Climate of the Past* **17**, 2223-2254 (2021).
22 Cheng, K. R., Andy; Hardisty, Dalton. Characterizing the marine iodine cycle and its relationship to ocean deoxygenation in an Earth System model. *EGUsphere [preprint]* (2024).
23 Sosdian, S. M. *et al.* Constraining the evolution of Neogene ocean carbonate chemistry using the boron isotope pH proxy. *Earth and Planetary Science Letters* **498**, 362-376 (2018).
