# Supplementary material for: Miocene ocean circulation shifted expansive oxygen deficient zones to the Atlantic
Source: Nat Commun. 2026 May 28;17:5935. doi: 10.1038/s41467-026-73732-7 (PMC13341793; doi:10.1038/s41467-026-73732-7)
Supplement: Supplementary file 2 — Transparent Peer Review file [file 41467_2026_73732_MOESM2_ESM.pdf]

Peer Review File

Miocene ocean circulation shifted expansive oxygen deficient zones to the Atlantic

Corresponding Author: Dr Dalton Hardisty

This file contains all reviewer reports in order by version, followed by all author rebuttals in order by version.

Version 0:

Reviewer comments:

Reviewer #1

(Remarks to the Author)
I appreciate the authors' efforts in addressing my previous comments. The manuscript has been significantly improved and clarified. I recommend publication in Nature Communications, once the authors have addressed the following issues:

Abstract

Line 14: Please use capital for “Ma”.

Main text

Line 55: I would recommend following the standard IODP terminoloy here and through the ms: refer to Site U1337 and Site U1338.

Line 90: Please use correct genus and species. Should be: Trilobatus sacculifer and Trilobatus trilobus.

Line 125: Plural needed for “Sites” here.

Line 233: Use acronym “MCO”.

Line 264: Please correct spelling for “PANGAEA” database.

Methods

Line 274: I think “sieved” may be more appropriate here.

Line 319: Capital needed for “Site” here.

Line 321: Please insert “Site” before “926”.

Lines 325-331: paragraph entitled “Sample Age and Paleolocation Determination”.
In their response to Reviewer’s 2 comments (see rebuttal), the authors stated: "Since the initial submission, updated age models have been published for these localities. As such, we have updated the age models for sites 321-1338, 321-1337, and 202-1237. This change is reflected in our Figure 2."
I note, however, that the text and cited references in this paragraph have NOT been revised in the updated manuscript. The text is identical to that in the originally submitted version. The authors do not mention that age models for Sites 1237, U1337 and U1338 have been updated and do not refer to the recent age model updates in Holbourn et al., 2024). Please revise this paragraph and the reference list accordingly.
Reference: Holbourn, A., Kuhnt, W., Kulhanek, D. K., Mountain, G., Rosenthal, Y., Sagawa, T., Lübbers, J., & Andersen, N.

(2024). Re-organization of Pacific overturning circulation across the Miocene Climate Optimum. Nature Communications, 15(1). https://doi.org/10.1038/s41467-024-52516-x

Supplemental Figures and Tables

Caption for Supplemental Fig.1: Isotope records from Site U1337 and U1338, but not from Site 1237, are included in the Westerhold et al., 2022 compilation shown in this figure. Reference to Holbourn et al., 2022 is not appropriate here, as it posdates Westerhold et al., 2020. Please amend the figure caption accordingly.

Supplemental Fig. 3: Please provide sample ID for the G. hexagonus specimens illustrated in this figure.

Supplemental Table 1: Only Top Depths are given. Please also provide Bottom Depths for all samples. This was previously requested by Reviewer 2 and you stated in the rebuttal that this table had been updated. This seems to have been overlooked during revision. Reviewer 2 also requested that original mbsf depths be provided for each sample.

Supplemental Table 1: Please note that mcd depths quoted for Sites U1337 and U1338 are in fact revised mcd depths. Please amend table accordingly.

Supplemental Table 4: Please use correct genus and species. Should be: Trilobatus trilobus.

Reviewer #2

(Remarks to the Author)
The authors examined the distribution of dissolved oxygen during the Miocene Climate Optimum (MCO), which was characterized by high atmospheric CO_2 concentrations and a warm climate, by reporting multi-species planktonic foraminiferal I/Ca records from three sites in the tropical Pacific and one site in the equatorial Atlantic. They observed a Pacific-Atlantic seesaw pattern of oxygen-depleted zones at the MCO, which is the opposite of the modern situation. Through Earth system model (eGENIE) simulations, the authors concluded that the seesaw pattern is related to the open Central American Seaway. Thus, the configuration of the continents had a stronger influence on ocean circulation and subsequent oxygen distribution than atmospheric CO_2 concentration.
The authors are pioneers in simulating oceanic iodine distribution. New records of geochemical and micropaleontological indicators covering the MCO are of great interest.
Despite these positive aspects, I have several concerns, particularly regarding the foraminiferal I/Ca data and its interpretation. I will address these concerns below.

1. Presentation and Interpretation of Planktonic Foraminiferal I/Ca
I am not convinced by the use of average I/Ca values for foraminifera (Figure 2). Figure S2 shows the I/Ca data for different species and provides more information because the observed scatter, particularly at ODP Site 1237, cannot be explained by differences in species alone. While showing a general trend with a red line is useful, presenting each data point would also be helpful.
The authors did not interpret the significant variability in I/Ca observed at ODP Site 1237 during the MCO. This variability appears to be real because the same trend was observed at ODP Site 1236 between 12.5 and 13 Ma by Nilsson-Kere et al. (2015). What are the possible reasons for this large variability? Could it be related to the proximity of the sites to the boundary of the oxygen-deficient zone (ODZ)? Or, could it be related to their proximity to the ODZ boundary, making them sensitive to slight changes in the boundary? An explanation is needed.
Finally, examining the planktonic foraminiferal I/Ca data only during the MCO did not demonstrate changes relative to the modern ocean. I appreciate Figure S16 and would like to see it in the main text. Similarly, I suggest acquiring data from recent sediments at ODP Site 926 to reinforce the interpretation. This is because low foraminiferal I/Ca values can be observed in oxygenated water (Figure S7). The authors used the abundance of G. hexagonus to support their interpretation, but its abundance is not solely related to oxygenation conditions. Demonstrating the difference between highly oxygenated water currently and potential oxygen depletion during the MCO would be more straightforward by comparing the foraminiferal I/Ca values in recent sediments at ODP Site 926.

2. Ambiguous Definition of ODZs
The authors used a threshold value of 75 µM to define ODZs in relation to a foraminiferal I/Ca value of 2.5 µmol/mol. However, they did not present the conversion of I/Ca into oxygen concentration. Additionally, the foraminiferal I/Ca threshold value of 2.5 µmol/mol may be subject to significant uncertainty, as more scattering has been observed with the accumulation of additional data since Lu et al.'s work in 2016. It would be easier to explain their definition of ODZs using a threshold oxygen concentration of 75 µM, which is higher than the general definition of an OMZ but represents biological stress. This definition should be indicated the first time ODZ is used.

Finally, the initial motivation of this study was to "draw lessons from the past regarding the impact of higher CO_2 and ocean warming on dissolved oxygen concentrations and marine ODZs." The main conclusion of this study is that continental configuration dominantly controls general ocean circulation. Does this mean that studies of the MCO cannot provide important information about future OMZ expansion? The authors mentioned the importance of accounting for continental configuration and ocean circulation. It would be interesting to include a brief statement about the contribution of this work to future studies.

I would suggest a major revision.

Below, I provide specific comments in the hope that the authors will find them helpful.

Minor/specific comments
Throughout the main text and supplementary information. Add a space between a number and a unit (ex. 500 ppm not 500ppm, 300 m not 300m etc.).

Throughout the main text and supplementary information. Indicate IODP sites such as "U1337" or "U1338." Distinguish ODP sites where "U" is unnecessary.

Throughout the main text and supplementary information. Add the year of publication after the author. For example, "Hess et al." on line 118 should be "Hess et al. (2025)."

Indicate the location of the marine sediments studied in this study, including water depths. This information is missing entirely.

Line 49: “falls below 2.5 µmol/mol”. While this value was indeed proposed as a threshold, its robustness is questionable when considering the scatter of foraminiferal I/Ca and oxygen concentrations shown in Figure S7. I would say foraminiferal I/Ca decreases and a threshold value of 2.5 µmol/mol was proposed.

Lines 51–52: “the foraminiferal I/Ca proxy…below low oxygen thresholds”. Some clarification is required. What did the authors mean by “an integrating signal” in this sentence? Did they envision a complex relationship between iodate and oxygen concentrations in relation to water mass mixing, the kinetic influence of changes in iodine speciation, or the incorporation processes into calcite tests? Please clarify. Was this "integrating" aspect considered in the iodate modeling?

Line 100: “G. hexagonus”. The name of a foraminifer should be fully spelled out the first time it is used.

Lines 107–108: “low I/Ca in this region…produced elsewhere in the ODZ”. This sentence is unclear. Did the authors mean that the iodate signal was transported so that the iodate concentration did not reflect the in situ oxygen concentration? If advection is an important factor in determining the I/Ca ratio of foraminifera, then using this proxy to estimate the in situ oxygenation state is questionable. Please clarify.

Line 119: ”sd” should spelled out.

Line 126: “exceptional preservation in Site 1338 compared to Site 1337 during our study interval”. Why is there a difference in preservation between the two sites? A brief explanation would be helpful.

Line 127: “Despite the differences, both cores maintain low I/Ca during the MCO”. Figure S16 clearly distinguishes the data from Nilsson-Kerr et al. (2025) and this study, but the sites are unclear. Please use different symbols to clarify.

Line 132-133: “abundant foraminiferal fragments”. I am confused by the evaluation of the preservation state. The text describes the abundance of fragmentation as an indicator, but the "Assessing Diagenetic Recrystallization" section and Table S3 only show SEM observations. Please be consistent.

Lines 195-196: Definition of the red lines. The red lines indicate 75 µM of O_2 based on their modeling. Their definition with foraminiferal I/Ca is confusing. Please revise it.

Lines 271-303: The "Foraminiferal Sample Preparation and Measurement" section. Was the Ca concentration aligned to a constant value, or was the Ca test not performed? What was the typical range of Ca concentrations for the samples and standard solutions?

Figures
Figure 2: Marking depth intervals where G. hexagonus was observed, along with the foraminiferal I/Ca data, will be helpful for ODP Site 962.

Figures 2 and 3. Which water depths of dissolved oxygen concentration are shown on the map? Please specify. Also, revise the caption concerning the red lines.

Supplementary information
Figure S1. The age scale direction differs between the entire Cenozoic era and the MCO zoom, which is confusing.

Figures S5, S6, S9, S10, and S11: Indicate the water depth of the distribution of iodate or oxygen concentrations shown in the maps.

Version 1:

Reviewer comments:

Reviewer #2

(Remarks to the Author)
I appreciate the authors' efforts to clarify the manuscript. Two new figures (Figures 2 and 4) largely contribute to the clarification.
As the manuscript has improved significantly, I only have a few suggestions.

Firstly, I recommend considering two recent publications (Wang et al., 2026; Guarinos et al., 2026). The first paper proposed a new threshold value of 2.3 µmol/mol for the foraminiferal I/Ca ratio, instead of 2.5 µmol/mol, with an associated dissolved oxygen concentration of approximately 90 µmol/kg. The second publication presents a new relationship between foraminiferal I/Ca ratios and oxygen concentration, examining the hypothesis proposed by Winkelbauer et al. (2023) that iodine is continuously acquired during the vertical settling of foraminiferal tests. These works would not affect the main message of the manuscript. However, considering these recent works could contribute to the longevity of the manuscript.

The second point concerns numerous typos and inconsistent formatting of archive and foraminiferal species names, etc. I have tried to indicate the corrections in the list of minor/specific comments below, but this list is not exhaustive. I would invite the authors to carefully read the text, figure captions, and supplementary information before final submission.

I would suggest accepting this work.

Minor/specific comments
Line 29: A space is necessary between "500" and "ppm".

Line 63: "pacific" should be start with a capital letter.

Lines 63 and 64, and throughout the text: Please use the standardized indication of archives collected with the ODP and IODP programs. The authors modified this point, but the current version combines expedition number and site in some cases. I suggest the following format: For example, instead of "IODP 320/321-U1337," write "IODP Expeditions 320 and 321, Site U1337" the first time it appears. Then, use "IODP Site U1337" in the following text, figure caption, and supplementary information. Incorrect notations are still found on the following lines: Lines 89, 97 (remove the expedition number "138"), and 107.

Lines 79, 80, 81, 97, 98 and throughout the text: Once the full name of a foraminiferal species has been presented, use the corresponding abbreviation in subsequent text, except at the beginning of a sentence.

Line 112: "and" should not be italic.

Lines 120 and 206 "Fig." should be replaced with "Figure."

Lines 120–122: It is unclear which data indicate that "Caribbean are well oxygenated throughout the water column". Please clarify.

Lines 148–150: the authors state that the excellent preservation of foraminiferal tests at Site U1338 is related to "the relatively high productivity and sedimentation rates." However, this explanation is confusing because a high organic matter content in the sediment could induce poor test preservation due to acidification. Please revise the text.

Lines 224 and 225: "the Atlantic Meridional Overturning Circulation". The authors may indicate "the Atlantic Meridional Overturning Circulation (AMOC)" here instead of on line 378.

Figure 4 legend: "Nilson-Kerr et al.," should be "Nilsson-Kerr et al.".

Supplemental
Lines 6 and 7: "delta" is missing before 13C and 18O.

References
Wang, X., Rickaby, R. E. M. & Lu, Z. A deep dive into the planktic foraminiferal I/Ca in global core-tops. Global Planet. Change 261, 105412 (2026). https://doi.org/https://doi.org/10.1016/j.gloplacha.2026.105412

Guarinos, V. et al. Planktonic foraminifera Iodine/Calcium ratio: is it a proxy for dissolved oxygen in the ocean? EGUsphere 2026, 1-24 (2026). https://doi.org/10.5194/egusphere-2026-622

Open Access This Peer Review File is licensed under a Creative Commons Attribution 4.0 International License, which permits use, sharing, adaptation, distribution and reproduction in any medium or format, as long as you give appropriate credit to the original author(s) and the source, provide a link to the Creative Commons license, and indicate if changes were made.
In cases where reviewers are anonymous, credit should be given to 'Anonymous Referee' and the source.
The images or other third party material in this Peer Review File are included in the article's Creative Commons license, unless indicated otherwise in a credit line to the material. If material is not included in the article's Creative Commons license and your intended use is not permitted by statutory regulation or exceeds the permitted use, you will need to obtain permission directly from the copyright holder.
To view a copy of this license, visit https://creativecommons.org/licenses/by/4.0/

REVIEWER COMMENTS

We thank the reviewers for their thoughtful feedback. Almost every revision requested has been accommodated or addressed in detail. We think the paper is greatly improved because of the these changes.

In particular, there are 3 main changes that reviewers requested that we think benefited the text:

1. We moved the species-specific I/Ca plot from the supplemement to the main text. It replaced the former Figure 2. This is a major improvement. Figure 2 clearly demonstrates to the reader that we measured I/Ca in from many of the same species across all sites, which makes these data more comparable. It is also more transparent about likely sources of variability. We have updated the text to more directly recognize and discuss the species-specific I/Ca results.
2. We have move the graph of Pacific I/Ca over last 17 Ma from the supplement to the main text (Figure 4). This is again an exciting addition, as our data fill gaps in this record and it allows us to discuss longer term trends. The text was also updated accordingly.
3. A clearer discussion of the iodate-oxygen relationship within the context of oxygen deficient zones. This was addressed by updating some of the intro and moving some text from later discussion to the introduction, thus allowing for this relationship to be explained earlier and thus less ambiguous during following discussion. We think this streamlines the text over all.

All comments are addressed below. The referenced line numbers refer to the version without track changes. If preferred, there is also a version submitted with track changes, but the line numbers will not align.

Reviewer #1 (Remarks to the Author):

I appreciate the authors' efforts in addressing my previous comments. The manuscript has been significantly improved and clarified. I recommend publication in Nature Communications, once the authors have addressed the following issues:

Thank you for the helpful feedback! The issues presented are now all addressed.

Abstract

Line 14: Please use capital for “Ma”.
Fixed

Main text

Line 55: I would recommend following the standard IODP terminoloy here and through the ms: refer to Site U1337 and Site U1338.

Fixed.

Line 90: Please use correct genus and species. Should be: Trilobatus sacculifer and Trilobatus trilobus.

Fixed.

Line 125: Plural needed for “Sites” here.

Fixed.

Line 233: Use acronym “MCO”.

Fixed.

Line 264: Please correct spelling for “PANGAEA” database.

Fixed.

Methods

Line 274: I think “sieved” may be more appropriate here.

Fixed.

Line 319: Capital needed for “Site” here.

Fixed.

Line 321: Please insert “Site” before “926”.

Fixed

Lines 325-331: paragraph entitled “Sample Age and Paleolocation Determination”.

In their response to Reviewer’s 2 comments (see rebuttal), the authors stated: "Since the initial submission, updated age models have been published for these localities. As such, we have updated the age models for sites 321-1338, 321-1337, and 202-1237. This change is reflected in our Figure 2."

I note, however, that the text and cited references in this paragraph have NOT been revised in the updated manuscript. The text is identical to that in the originally submitted version. The authors do not mention that age models for Sites 1237, U1337 and U1338 have been updated and do not refer to the recent age model updates in Holbourn et al., 2024). Please revise this paragraph and the reference list accordingly.

Reference: Holbourn, A., Kuhnt, W., Kulhanek, D. K., Mountain, G., Rosenthal, Y., Sagawa, T., Lübbers, J., & Andersen, N. (2024). Re-organization of Pacific overturning circulation across the Miocene Climate Optimum. Nature Communications, 15(1). https://doi.org/10.1038/s41467-024-52516-x

Thanks for pointing out this oversight. Yes, the updated age models were incorporated into our figures, but we did not provide the new citation. This is now provided in the

"Sample Age and Paleolocation Determination" section.

Supplemental Figures and Tables

Caption for Supplemental Fig.1: Isotope records from Site U1337 and U1338, but not from Site 1237, are included in the Westerhold et al., 2022 compilation shown in this figure. Reference to Holbourn et al., 2022 is not appropriate here, as it posdates Westerhold et al., 2020. Please amend the figure caption accordingly.

Thanks for this comment. We are a bit confused about the comment details but upon scrutiny of the figure, we found that the citations from the d13C graphs from Holbourn et al., 2007 and Holbourn et al., 2015 are incorrectly cited in the figure caption. We have now corrected the figure caption citations accordingly.

Supplemental Fig. 3: Please provide sample ID for the G. hexagonus specimens illustrated in this figure.
This is now provided in the caption to this figure.

Supplemental Table 1: Only Top Depths are given. Please also provide Bottom Depths for all samples. This was previously requested by Reviewer 2 and you stated in the rebuttal that this table had been updated. This seems to have been overlooked during revision. Reviewer 2 also requested that original mbsf depths be provided for each sample.
We have provided the bottom depths now.

Supplemental Table 1: Please note that mcd depths quoted for Sites U1337 and U1338 are in fact revised mcd depths. Please amend table accordingly.
We have now updated "Sample Age and Paleolocation Determination" section with the proper citations and added a reference to it in the Caption for Supplementary Table 1 for sources of age estimates.

Supplemental Table 4: Please use correct genus and species. Should be: Trilobatus trilobus.
Thanks. Fixed.

Reviewer #2 (Remarks to the Author):

The authors examined the distribution of dissolved oxygen during the Miocene Climate Optimum (MCO), which was characterized by high atmospheric CO_2 concentrations and a warm climate, by reporting multi-species planktonic foraminiferal I/Ca records from three sites in the tropical Pacific and one site in the equatorial Atlantic. They observed a Pacific-Atlantic seesaw pattern of oxygen-depleted zones at the MCO,

which is the opposite of the modern situation. Through Earth system model (eGENIE) simulations, the authors concluded that the seesaw pattern is related to the open Central American Seaway. Thus, the configuration of the continents had a stronger influence on ocean circulation and subsequent oxygen distribution than atmospheric CO_2 concentration.

The authors are pioneers in simulating oceanic iodine distribution. New records of geochemical and micropaleontological indicators covering the MCO are of great interest.

Despite these positive aspects, I have several concerns, particularly regarding the foraminiferal I/Ca data and its interpretation. I will address these concerns below.

Thank you for the helpful feedback! The issues presented are now all addressed. As outlined below, we feel strongly that the inclusion of the species specific I/Ca MCO data and the larger temporal I/Ca record to the main text are major improvements and are grateful for the input and requests.

1. Presentation and Interpretation of Planktonic Foraminiferal I/Ca

I am not convinced by the use of average I/Ca values for foraminifera (Figure 2). Figure S2 shows the I/Ca data for different species and provides more information because the observed scatter, particularly at ODP Site 1237, cannot be explained by differences in species alone. While showing a general trend with a red line is useful, presenting each data point would also be helpful.

We have now plotted the species-specific I/Ca in Figure 2 of the main text and included it below. We agree that this is more transparent and informative in multiple ways. For example, this clarifies to the reader that:

1. *D. altispira* and *D. venezuelana* were measured in all sample sets, including Site 845 from Hess et al., 2023.
2. That site 926, our Atlantic Site with low I/Ca, included a large diversity of foraminfera, including those also measured and shown to have the potential to have high I/Ca values (e.g., *D. altispira* and *D. venezuelana*). This reinforces the I/Ca interpretation of low oxygen at this site, as also supported by the model as opposed to species-specific issues.

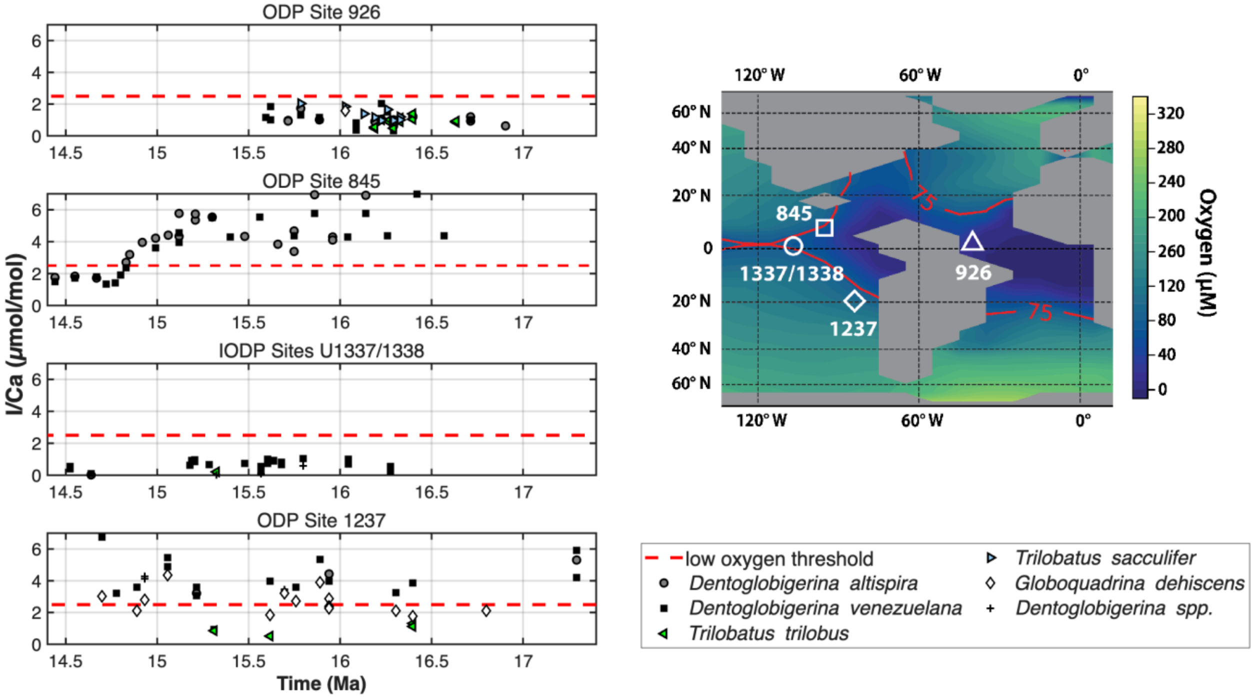

The authors did not interpret the significant variability in I/Ca observed at ODP Site 1237 during the MCO. This variability appears to be real because the same trend was observed at ODP Site 1236 between 12.5 and 13 Ma by Nilsson-Kere et al. (2015). What are the possible reasons for this large variability? Could it be related to the proximity of the sites to the boundary of the oxygen-deficient zone (ODZ)? Or, could it be related to their proximity to the ODZ boundary, making them sensitive to slight changes in the boundary? An explanation is needed.

We agree and now added the following text at Line 73. Importantly, in addition to comparison to the Nilsson-Kerr et al., data, the suggested addition of the updated Figure 2 to display species-specific I/Ca helps strengthen this interpretation.

"Foraminifera from ODP Site 1237—our southernmost Pacific site—were measured between 17.3-14.8 Ma. Overall, the I/Ca variability from ODP 1237 indicate that the locality was likely near the fringe but not directly within low oxygen waters characterizing an ODZ. Species measured from this site, chosen based on abundance and preservation, were *Dentoglobigerina venezuelana, Dentoglobigerina altispira, Globoquadrina dehiscens,* and, less frequently, *Trilobatus trilobus.* Throughout most of this interval, the average I/Ca values of foraminiferal specimens were above low oxygen thresholds (Figure 2), but there are species-specific I/Ca differences not observed at other sites. Specifically, *Dentoglobigerina venezuelana* and *Dentoglobigerina altispira* are elevated, but variable, while *Globoquadrina dehiscens* oscilates near the low oxygen I/Ca threshold. A smaller pool of samples from *Trilobatus trilobus* remain within I/Ca ranges interpreted as representing low oxygen waters. These variable I/Ca MCO observations at ODP Site 1237 are supported in part by similar I/Ca trends observed from an independent Site 1237 study bookending the MCO[28]. The intraspecies variations may represent temporal fluctuations in proximity to low oxygen waters. Interspecies differences may represent variability within depth habit and thus proximity to low oxygen waters or

currently uncharacterized species-specific partitioning of iodate. Consistent with this interpretation, the paleolocation of the ODP Site 1237 places it further from the direct site of marginal upwelling during the MCO relative to today[29]. While dissolved iodate is lowest in modern marginal upwelling localities, low iodate (high iodide) waters do persist offshore in the modern Pacific (Supplementary Figure 3), thus allowing the potential for ODP-202-1237 to track ODZ distribution via foraminiferal I/Ca and increasing its value as a comparison to the modern."

Finally, examining the planktonic foraminiferal I/Ca data only during the MCO did not demonstrate changes relative to the modern ocean. I appreciate Figure S16 and would like to see it in the main text.
We agree. This is a great addition and rich data set to incorporate with. It has now been cleaned up and added to the main text as Figure 4. It is also pasted below for context.

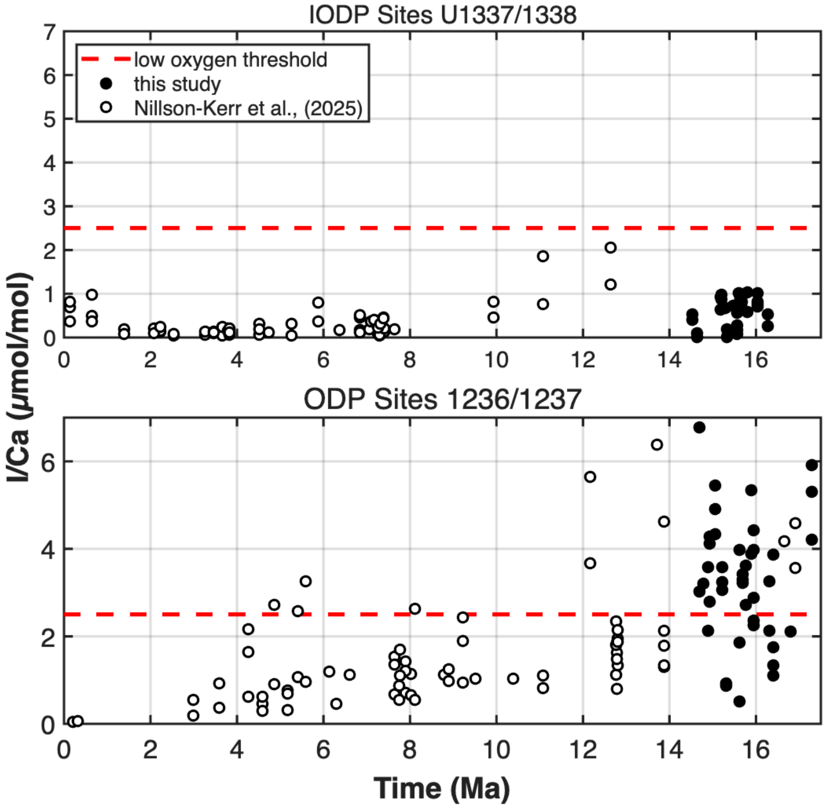

Similarly, I suggest acquiring data from recent sediments at ODP Site 926 to reinforce the interpretation. This is because low foraminiferal I/Ca values can be observed in oxygenated water (Figure S7). The authors used the abundance of G. hexagonus to support their interpretation, but its abundance is not solely related to oxygenation conditions. Demonstrating the difference between highly oxygenated water currently and potential oxygen depletion during the MCO would be more straightforward by comparing the foraminiferal I/Ca values in recent sediments at ODP Site 926.

We are unable to do obtain new data from Site 926 modern sediments for financial and logistical reasons. The postdoc leading the project has started a new non-research job and the funds for this project are expired.

That said, we don't believe it is necessary to obtain new coretop I/Ca data for the following reasons:

1. There are already an abundance of I/Ca data from the Atlantic. In order to demonstrate this and that there are no strange Atlantic-specific anomalies, we separated out the Atlantic core top data in the modern coretop compilation figure in the supplement (Supplementary Figure 2), which is also pasted below. In summary, the Atlantic data follow the general trend seen elsewhere, with low I/Ca becoming exclusive near 75 µmol/kg O2, the threshold we use for the proxy in this paper.
2. Similar species, in particular *Dentoglobigerina venezuelana* and *Dentoglobigerina altispira* are measured at all 4 sites and are low at Site 926 and U1337/1338 but are elevated at Sites 845 and 1237, indicating that the low I/Ca values at Site 926 are most likely related to its vicinity to low oxygen waters and not species-specific iodate incorporation that is anomalously low.
3. The New Figure 2 also demonstrates that a wide diversity of foraminifera were measured for I/Ca at Site 926 and all return low values.

We updated the text at line 107 (pasted below) to reflect the points above:

"Foraminifera from the western Atlantic (ODP Site 124-926) also record I/Ca values that indicate that the site had an expanded ODZ throughout the observed interval, ~15.5-16.9 Ma. A diversity of species were measured from these samples, all with low I/Ca values, including the surface dwelling *Trilobatus sacculifer* and *Trilobatus trilobus* as well as some of the intermediate/deep dwelling *Dentoglobigerina venezuelana* and intermediate dwelling *Globoquadrina dehiscens* and *Dentoglobigerina altispira*. Importantly, *Dentoglobigerina venezuelana and Dentoglobigerina altispira* were also measured at each other site and show the potential to have elevated I/Ca at ODP Sites 1237 and 845, indicating that the low values at ODP Site 926 are not related to a species-specific vital effect. In addition, foraminiferal I/Ca in the modern Atlantic show a similar relationship to O_2 as other ocean basins (Supplementary Figure 2), supporting that our observations reflect foraminiferal proximity to low oxygen waters as opposed to a currently uncharacterized Atlantic-specific basinal effect on foraminiferal I/Ca."

Further, we continue to use *Globorotaloides hexagonus* to support our interpretation but have softened the wording to emphasize this observation is not the main driver of our interpretation. Importantly, we don't rely solely on *G. hexagonus* for our Atlantic OMZ interpretation. It is really the low I/Ca and the independent model that best support low oxygen conditions in the western Atlantic.

New text for *G. hexagonus* at line 124:

“For example, the presence of *Globorotaloides hexagonus* has been demonstrated to be most abundant in areas that have strong midwater ODZs and thus its presence in ancient sediments has been applied as an indicator of the presence of low oxygen water column conditions during burial[28,35,36]. At ODP Site 926, we observed the presence of specimens of *G. hexagonus* in every one of a subset of nine samples examined, although those specimens were not included in the I/Ca measurements (Supplemental Figure 4; Supplemental Table 1).”

2. Ambiguous Definition of ODZs

The authors used a threshold value of 75 µM to define ODZs in relation to a foraminiferal I/Ca value of 2.5 µmol/mol. However, they did not present the conversion of I/Ca into oxygen concentration.

We interpret I/Ca has having a threshold value for depletion that is related to oxygen (while clarifying that this threshold is approximate and requires further research), but do not support a direct relationship that allows conversion of I/Ca values to O2 values. No other work has done this either. It is also clear from our Supplementary Figure 2 that low I/Ca values become exclusive at O2 values somewhere near 75 µM, but that variability beyond this may not solely be related to oxygen. For the above reasons and to remain conservative in our interpretations, we did not convert I/Ca to O2.

Additionally, the foraminiferal I/Ca threshold value of 2.5 µmol/mol may be subject to significant uncertainty, as more scattering has been observed with the accumulation of additional data since Lu et al.'s work in 2016. It would be easier to explain their definition of ODZs using a threshold oxygen concentration of 75 µM, which is higher than the general definition of an OMZ but represents biological stress. This definition should be indicated the first time ODZ is used.

We agree with this uncertainty and have made this clear through the addition of the following text at Line 51, so now we are clarifying the I/Ca-O2 relationship early in the text:

“Importantly, the I/Ca proxy most closely tracks the minimum O_2 in underlying or adjacent waters as opposed to the O_2 concentrations in waters directly hosting the foraminiferal life cycle. This relationship between I/Ca and dissolved minimum O_2 in the water column is strong in core-top samples[16] but the exact timing of iodate incorporation into foraminiferal calcite has yet to be determined[21] and could be impacted by post-mortem or post-depositional burial processes. This indicates that the foraminiferal I/Ca proxy is an integrated signal across the water column, incorporating subsurface waters above and below low oxygen thresholds[21]. An I/Ca range of <2.5 µmol/mol is herein and commonly assigned to O_2 values <75 µM, but this I/Ca-O_2 threshold relationship is subject to uncertainty (Supplementary Figure 2)[8,20,22-24]. The proxy nonetheless remains useful because open ocean oxygen concentrations in this range are specific to ODZ-linked water masses imparting biological stress[25].”

Finally, the initial motivation of this study was to "draw lessons from the past regarding the impact of higher CO_2 and ocean warming on dissolved oxygen concentrations and marine ODZs." The main conclusion of this study is that continental configuration

dominantly controls general ocean circulation. Does this mean that studies of the MCO cannot provide important information about future OMZ expansion? The authors mentioned the importance of accounting for continental configuration and ocean circulation. It would be interesting to include a brief statement about the contribution of this work to future studies.

Yes, we think a take home is that multiple high-profile studies are overinterpreting Cenozoic warming events, including the MCO, as analogues to the modern. In the case of the MCO, the Hess et al., (2023) paper used observations from the Pacific to make broader generalizations about ODZ response to warming, in this case linking warming to the smaller MCO Pacific ODZ. These studies use more limited I/Ca data than us and also lack the models. Our expanded dataset and model demonstrate that the limited ODZ in the Pacific was unlikely due to the warming conditions of the MCO and more related to ocean circulation, and therefore recent studies claiming warming will shrink ODZs based on MCO Pacific data are underdeveloped. To be more explicit about this, we separated out a final paragraph in the conclusions that is more straightforward, which is pasted below from Line 269:

"Our joint Atlantic and Pacific observations and model indicate that interpretations of warming-induced ODZ contraction drawn from the Pacific during the MCO[8,13] may be incomplete analogues for comparison to ODZs during modern and future warming. Ultimately, while Cenozoic warm periods—including the MCO—can inform future impacts of continuing carbon emissions and warming, it is essential to account for differences in continental configuration and ocean circulation that impact local and global ODZ distribution."

I would suggest a major revision.

Below, I provide specific comments in the hope that the authors will find them helpful.

Minor/specific comments

Throughout the main text and supplementary information. Add a space between a number and a unit (ex. 500 ppm not 500ppm, 300 m not 300m etc.).

Fixed.

Throughout the main text and supplementary information. Indicate IODP sites such as "U1337" or "U1338." Distinguish ODP sites where "U" is unnecessary.

Fixed.

Throughout the main text and supplementary information. Add the year of publication after the author. For example, "Hess et al." on line 118 should be "Hess et al. (2025)."

We have not updated this because the citation is provided as a superscript in each case and our current formatting follows Nature Communication guidelines.

Indicate the location of the marine sediments studied in this study, including water depths. This information is missing entirely.

Now provided as Supplementary Table 6 and pasted below.

STable 6. Modern and paleo coordinates for study locations. Also includes modern water depths. Details for source of paleocoordinates provided in the "Sample Age and Paleolocation Determination" section of main text.

Site	Modern Latitude (°N)	Paleo-Latitude (°N)	Modern Longitude (°W)	Paleo-Longitude (°W)	Modern water depth (m)
IODP 320-U1337	2.3	0.2133	-117.58	-111.0398	4,464
ODP 202-1237	16.04	-17.43	-76.11	-83.29	3,212
ODP 138-845	9.34	5.608	-94.35	-99.543	3,704
ODP 154-926	3.7	3.575	-42.9	-41.344	3,610

Line 49: “falls below 2.5 μmol/mol”. While this value was indeed proposed as a threshold, its robustness is questionable when considering the scatter of foraminiferal I/Ca and oxygen concentrations shown in Figure S7. I would say foraminiferal I/Ca decreases and a threshold value of 2.5 μmol/mol was proposed.
We have added additional context in response to a previous comment. See response to the related previous comment and text at Line 51.

Lines 51–52: “the foraminiferal I/Ca proxy…below low oxygen thresholds”. Some clarification is required. What did the authors mean by “an integrating signal” in this sentence? Did they envision a complex relationship between iodate and oxygen concentrations in relation to water mass mixing, the kinetic influence of changes in iodine speciation, or the incorporation processes into calcite tests? Please clarify. Was this "integrating" aspect considered in the iodate modeling?
“Integrated” means that planktic I/Ca values seem to be more closely related to the vertical minimum O2 values in the water column than the O2 in water in which they live.

We have now clarified this in the introduction at Line 51 in the same location we discussed the I/Ca-O2 relationship.

Line 100: “G. hexagonus”. The name of a foraminifer should be fully spelled out the first time it is used.
Done.

Lines 107–108: “low I/Ca in this region…produced elsewhere in the ODZ”. This sentence is unclear. Did the authors mean that the iodate signal was transported so that the iodate concentration did not reflect the in situ oxygen concentration? If advection is an important factor in determining the I/Ca ratio of foraminifera, then using this proxy to estimate the in situ oxygenation state is questionable. Please clarify.

Yes, this is what we mean. We provide a detailed section in Line 305 (pasted below) explaining this relationship. In summary, we disagree that this means the proxy is questionable to estimate in situ oxygenation but agree that these details should be considered during interpretation. Ultimately, low iodate and O2 are correlated. However, as we note in the text pasted below, at the bottom and outer fringe of an OMZ, iodate increases faster than O2, meaning that you can start to get high iodate while O2 remains relatively low. Our model accounts for iodate/O2 advection, reduction, oxidation, and biological cycling, but we note, as shown in Cheng et al., 2024, the model performs most poorly at reproducing in situ iodate at the bottom of and outer regions of ODZs, where both the spatial resolution of the model is limited and the uncertainties of the relationships of combinations of iodide oxidation and mixing with adjacent water masses are most pronounced.

Line 179:

"The modeled iodate concentrations indicate that the iodate reducing region within the Pacific ODZ is more constricted relative to the modeled low O_2 region and does not extend westward to IODP Sites U1337/1338 where low I/Ca is observed (Supplementary Figure 6 and 7). That our modelling does not reproduce the low iodate region relative to that identified via I/Ca might simply be a consequence of the models relatively low spatial resolution (10° in longitude can ca. 5° in latitude close to the Equator) (e.g., see Cheng et al.[26] for a discussion). Another possible explanation for the difficulty in resolving the sharp spatial I/Ca gradient between the Sites U1337/1338 and 845 via the model iodate is uncertainty in our understanding of the iodate-O_2 relationship in modern settings. This is particularly relevant along ODZ boundaries. For example, modern observations indicate that higher iodate concentrations are present before similar increases in [O_2] along the ODZ margins, including both laterally and at the lower oxycline of the ODZ. Instead of a strict linear relationship, iodate-oxygen can be best explained by active iodate reduction constrained to productive, marginal upwelling zones hosting denitrification, followed by subsequent internal-ODZ transport and preservation of these low iodate waters beyond regions of active iodate reduction (Supplementary Figures 2 and 3). Iodate concentrations eventually increase at these offshore ODZ margins prior to similar O_2 increases because of the combination of iodide oxidation and mixing with adjacent high iodate water masses (Supplementary Figure 3)[30,31,43,46]. Thus, while our data-model comparison generally supports the observations of a restricted Pacific ODZ relative to today, there is uncertainty in resolving the modeled ODZ boundary at the high spatial resolution of our foraminiferal I/Ca data."

Line 119: "sd" should spelled out.
This is removed since we no longer show the average and standard deviation in Figure 2, but instead show the species specific I/Ca, which we think improves the paper.

Line 126: "exceptional preservation in Site 1338 compared to Site 1337 during our study interval". Why is there a difference in preservation between the two sites? A brief explanation would be helpful.
Good question. The preservation differences between Site U1338 and U1337 are well documented within the provided citation and verified here, and explanation for why is warranted. We have now provided the following at Line 148:

"Observed preservational differences have been linked to the relatively higher productivity and sedimentation rates at Site U1338 limiting the residence time of foraminifera within stages of more corrosive diagenetic conditions[44]."

Line 127: "Despite the differences, both cores maintain low I/Ca during the MCO". Figure S16 clearly distinguishes the data from Nilsson-Kerr et al. (2025) and this study, but the sites are unclear. Please use different symbols to clarify.
This figure has been updated and moved to the main text, as requested by the reviewer. The updates deem that this comment is no longer relevant.

Line 132-133: "abundant foraminiferal fragments". I am confused by the evaluation of

the preservation state. The text describes the abundance of fragmentation as an indicator, but the "Assessing Diagenetic Recrystallization" section and Table S3 only show SEM observations. Please be consistent.

We agree with the reviewer that our assessment of preservation here is inconsistent with our later description using SEM. We did observe more fragments from Site 1237, but the observation is qualitative and analysis relatively incomplete. In agreement with the reviewers comments, we have removed this reference to “abundant “foraminiferal fragments” from the main text.

Lines 195-196: Definition of the red lines. The red lines indicate 75 µM of O_2 based on their modeling. Their definition with foraminiferal I/Ca is confusing. Please revise it.

We have updated the text in the intro to clarify the I/Ca-O2 relationship. See comments above and text at Line 51.

Lines 271-303: The "Foraminiferal Sample Preparation and Measurement" section. Was the Ca concentration aligned to a constant value, or was the Ca test not performed? What was the typical range of Ca concentrations for the samples and standard solutions?

The samples and standards were matrix matched at 50 ppm Ca. This is the common approach for I/Ca first proposed by Lu et al., 2010 and has been tested and confirmed in subsequent studies, including in our lab. To clarify this, we made the following addition at Line 316:

“Each standard contained 50 ppm Ca, and samples were diluted to 50 ppm Ca to matrix match to the standards.”

Figures

Figure 2: Marking depth intervals where G. hexagonus was observed, along with the foraminiferal I/Ca data, will be helpful for ODP Site 962.

We added this information now to the of Supplementary Table 1. We elected to not add it to the figure because not all samples were evaluated for *G. hexagonus* so adding in the data there gives the perhaps false perception that there are not *G. hexagonus* in some intervals we measured for I/Ca.

Figures 2 and 3. Which water depths of dissolved oxygen concentration are shown on the map? Please specify. Also, revise the caption concerning the red lines.

These are now provided in the supplement and copy and pasted below.

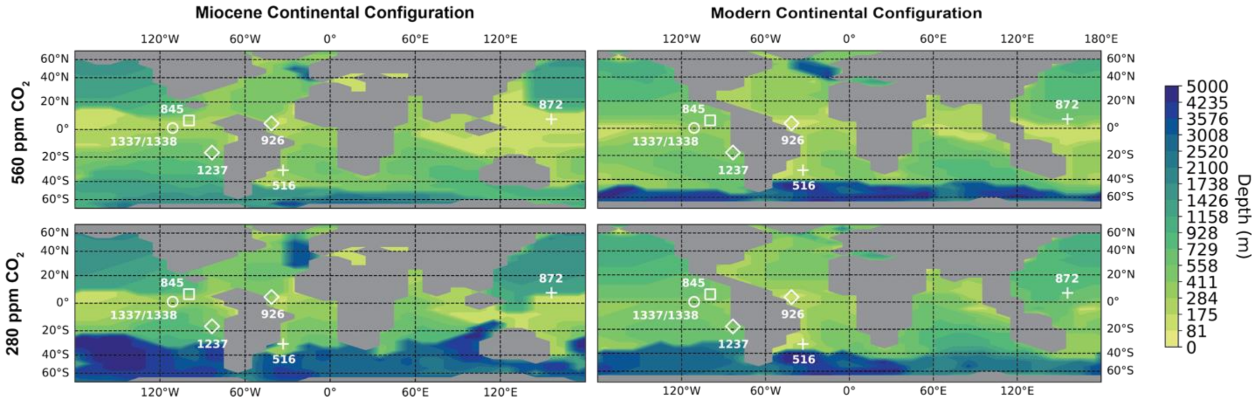

SFigure 15. Corresponding depths of minimum oxygen and associated iodate (or I/Ca) from Main Text Figure 2, Main Text Figure 3, Supplementary Figure 6, and Supplementary Figure 7.

Supplementary information

Figure S1. The age scale direction differs between the entire Cenozoic era and the MCO zoom, which is confusing.

Fixed.

Figures S5, S6, S9, S10, and S11: Indicate the water depth of the distribution of iodate or oxygen concentrations shown in the maps.

These are now provided in the Supplement Figures 15-17 and copy and pasted below (Supplementary 15 is pasted just above). The supplement now has the corresponding depth of minimum O2 and corresponding iodate (I/Ca) from all model simulations provided in the main text or supplement.

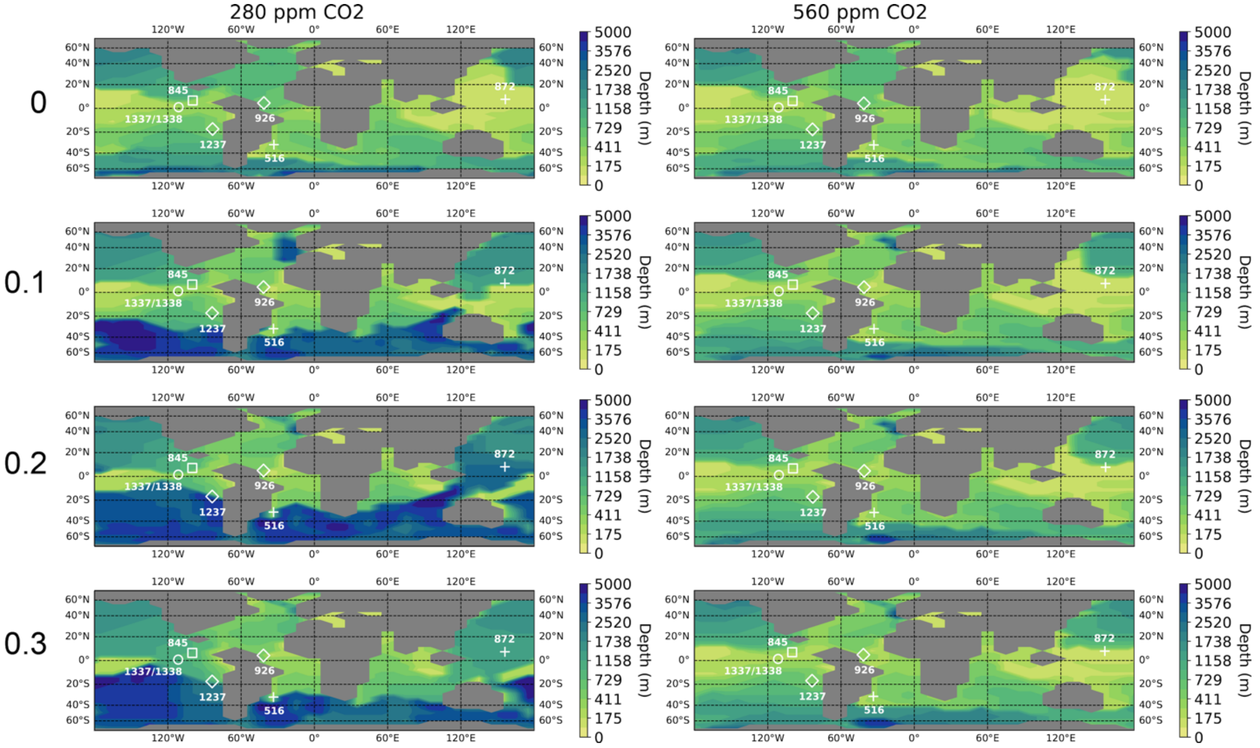

SFigure 16. **Corresponding depths of minimum oxygen and corresponding iodate (or I/Ca) from Supplementary Figure 8 and Supplementary Figure 10.**

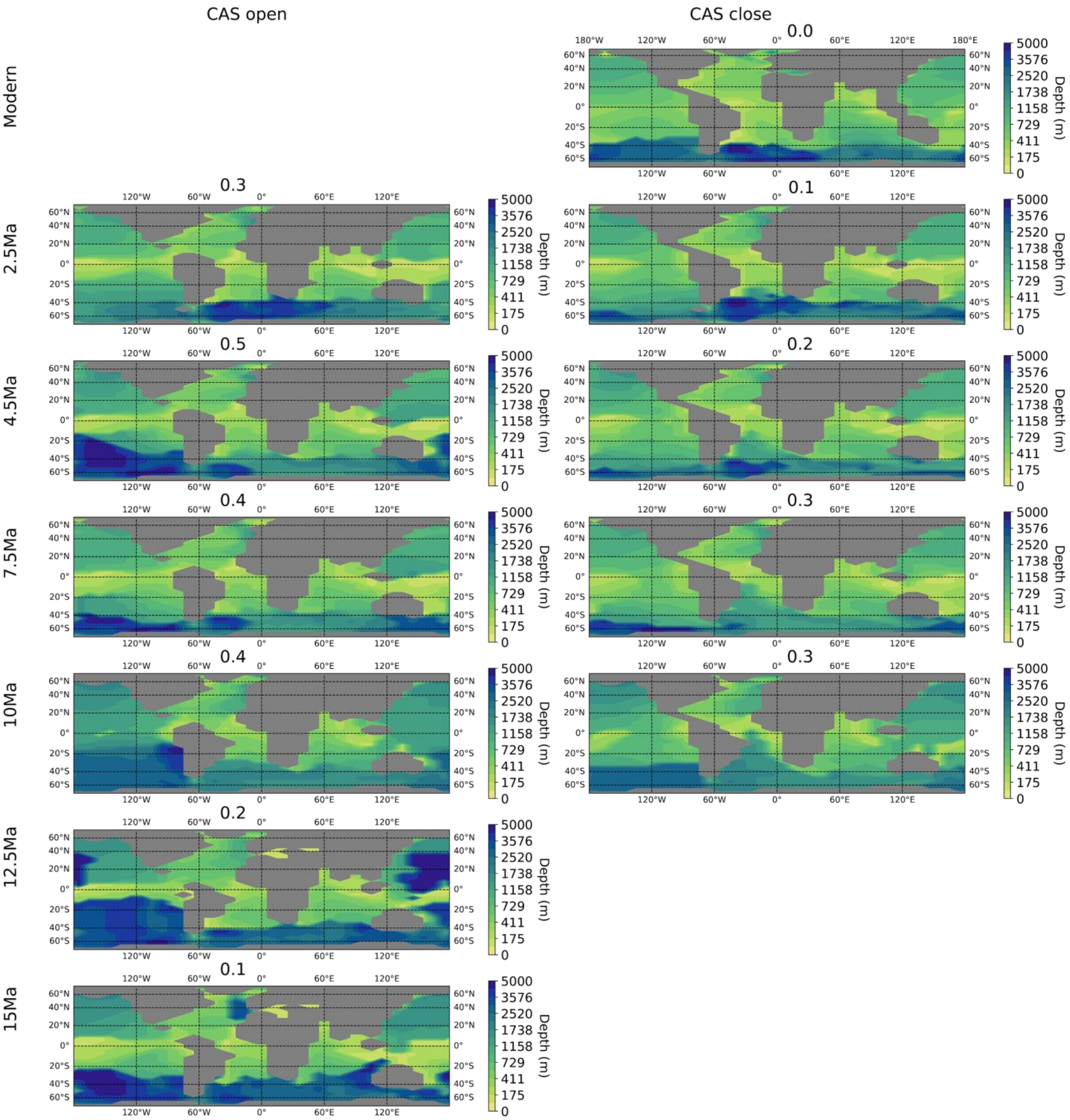

SFigure 17. **Corresponding depths of minimum oxygen from Supplementary Figure 9.**

Firstly, I recommend considering two recent publications (Wang et al., 2026; Guarinos et al., 2026). The first paper proposed a new threshold value of 2.3 μmol/mol for the foraminiferal I/Ca ratio, instead of 2.5 μmol/mol, with an associated dissolved oxygen concentration of approximately 90 μmol/kg. The second publication presents a new relationship between foraminiferal I/Ca ratios and oxygen concentration, examining the hypothesis proposed by Winkelbauer et al. (2023) that iodine is continuously acquired during the vertical settling of foraminiferal tests. These works would not affect the main message of the manuscript. However, considering these recent works could contribute to the longevity of the manuscript.

The citation to Wang et al., 2026 has been added and the I/Ca threshold updated to 2.3 μmol/mol in both the main text and in Figures 2 and 4 of the main text (red dashed line in both). As the reviewer indicates, this imparts no change in our interpretations. The 75 μM O2 threshold for I/Ca changes has not been updated but the Wang paper proposing a 90 μM O2 value is now cited in a line (quoted below from line 58) in the same sentence noting the uncertainty around this value. We did not change the value because there is uncertainty, and we did not wish to make changes to the contours in Figures 2 and 3 at this stage of review.

"An I/Ca range of <2.3 μmol/mol is herein and commonly assigned to O_2 values <75 μM, but this I/Ca-O_2 threshold relationship is subject to uncertainty (Supplementary Figure 2)[8,20,23-26]."

We did not add citation to Guarinos et al., 2026 because this is actually a preprint still under review and the reviews (available online with the preprint, which our co-author Dalton Hardisty was one) recommend major revisions which will impact outcomes of the paper relative to its current form.

The second point concerns numerous typos and inconsistent formatting of archive and foraminiferal species names, etc. I have tried to indicate the corrections in the list of minor/specific comments below, but this list is not exhaustive. I would invite the authors to carefully read the text, figure captions, and supplementary information before final submission.

We have corrected all suggestions below and carefully read through the text to find additional minor typos/inconsistencies.

I would suggest accepting this work.

Minor/specific comments

Line 29: A space is necessary between "500" and "ppm".

fixed

Line 63: "pacific" should be start with a capital letter.

Fixed

Lines 63 and 64, and throughout the text: Please use the standardized indication of archives collected with the ODP and IODP programs. The authors modified this point, but the current version combines expedition number and site in some cases. I suggest the following format: For example, instead of "IODP 320/321-U1337," write "IODP Expeditions 320 and 321, Site U1337" the first time it appears. Then, use "IODP Site U1337" in the following text, figure caption, and supplementary information. Incorrect notations are still found on the following lines: Lines 89, 97 (remove the expedition number "138"), and 107.

Fixed throughout according to suggestion.

Lines 79, 80, 81, 97, 98 and throughout the text: Once the full name of a foraminiferal species has been presented, use the corresponding abbreviation in subsequent text, except at the beginning of a sentence.

Fixed throughout according to suggestion.

Line 112: “and” should not be italic.

Fixed.

Lines 120 and 206 "Fig." should be replaced with "Figure."

Fixed.

Lines 120–122: It is unclear which data indicate that “Caribbean are well oxygenated throughout the water column”. Please clarify.

Clarified to “modern” and “generally” since this characterizes the Caribbean broadly today. See Line 117 pasted below.

“The modern western Atlantic and Caribbean are generally well oxygenated throughout the water column and Atlantic ODZ dissolved iodate concentrations generally do not reach the low levels observed in the more reducing waters of the Pacific ODZs (Supplementary Figure 2)[35,36].”

Lines 148–150: the authors state that the excellent preservation of foraminiferal tests at Site U1338 is related to "the relatively high productivity and sedimentation rates." However, this explanation is confusing because a high organic matter content in the sediment could induce poor test preservation due to acidification. Please revise the text.

We agree and so do the authors we cited as the original interpretation. The important component is the high sediment accumulation rates, which means deposited forams spend less time in the sediment layers with these acidic conditions. We have updated the line (now line 138) to the below to clarify. The replacement of “productivity and sedimentation” with “depositional” merges these themes into one concept, which we hope simplifies the interpretation that high sediment accumulation limits the time forams spend in the acidic or corrosive sediment layers the reviewer refers to.

“Observed preservational differences have been linked to the relatively higher depositional rates at IODP Site U1338 limiting the residence time of foraminifera within sediment layers with more corrosive diagenetic conditions[46].”

LLines 224 and 225: “the Atlantic Meridional Overturning Circulation”. The authors may indicate “the Atlantic Meridional Overturning Circulation (AMOC)” here instead of on line 378.

Fixed. Substituted later text with “AMOC”.

Figure 4 legend: “Nilson-Kerr et al.,” should be “Nilsson-Kerr et al.”.

Fixed

Supplemental

Lines 6 and 7: “delta” is missing before 13C and 18O.

Fixed.
